# Eurogame escape room of physics in veterinary education: a pilot study

**DOI:** 10.1186/s12917-025-05186-w

**Published:** 2025-12-20

**Authors:** Sascha Albert Bräuninger, Damian Alexander Motz, Matthias Lüpke, Hermann Seifert

**Affiliations:** 1 Institute of General Radiology and Medical Physics, University of Veterinary Medicine Hannover Foundation, Bischofsholer Damm 15, 30173 Hannover, Lower Saxony Germany; 2 Institute of Sanitary Engineering and Waste Management, Leibniz University Hannover, Welfengarten 1, 30167 Hannover, Lower Saxony Germany

**Keywords:** Escape room of physics, Gamification, European board games, Eurogames, Victory points

## Abstract

Physics education of students in disciplines in which physics is a minor subject faces various challenges such as the frequent students’ perception as difficult and abstract, and the often limited intrinsic motivation and engagement of minor-subject students. Gamification and game-based learning methods, such as educational escape rooms, are discussed as potential solution approaches. We present the concept of an educational escape room for the physics education of minor-subject students. The concept was developed as a hybrid between a classical educational escape room with sequential physics puzzles and the motivational elements of modern European board games (*Eurogames*). For example, this includes a corresponding victory point system and optional puzzles (called *Eurogame puzzles)* to provide individual paths for players. In the context of a first pilot study, the concept was designed for and evaluated with students of veterinary medicine and aims to increase both engagement and knowledge retention. An advantage is the flexibility of choosing from a broad range of optional puzzles to fascinate and motivate students, taking into account individual preferences and improving educational escape rooms by decision-making skills. We have addressed the two questions of (i) the acceptance of the concept by the students and (ii) the increase in knowledge of the students. To answer (i), the students anonymously completed a feedback questionnaire to communicate their personal opinion of the elements of the concept. To answer (ii), an approach of 12 physics questions was chosen by testing puzzle-associated factual knowledge, neglecting possible beneficial increases in experimental skills or reduction in physics anxiety for the present, through a pre- and posttest. Both, (i) and (ii), could be answered positively. The concept was successfully and gratefully accepted by the students, while the learning gain was measurable.

**Supplementary Information:**

The online version contains supplementary material available at 10.1186/s12917-025-05186-w.

## Background

Escape rooms have emerged as an innovative educational tool that offers students the opportunity to solve active team-based problems under time constraints [1–5]. A comprehensive meta-analysis indicates that educational escape rooms serve as exceptionally effective learning tools, as evidenced by their widespread implementation and reported success in enhancing educational experiences [6]. In general, an escape room is

a live-action team-based game where players discover clues, solve puzzles, and accomplish tasks in one or more rooms in order to accomplish a specific goal (usually escaping from the room) in a limited amount of time [7].

Although educational escape rooms, in the context of game-based learning approaches, are becoming popular in higher education [8], especially in medical and biological training, examples in physics and interdisciplinary groups remain rare [9]. Meanwhile, the popularity of European board games, which emphasize victory point systems, strategic choices, and player engagement, continues to grow [10] and potential applications of these games in education are also discussed. For example, in astrophysics, selected Eurogames are discussed to have good scientific accuracy for educational purposes [11, 12]. Physics is often perceived as abstract and challenging by students of non-physics disciplines, more precisely, of disciplines in which physics is usually just a minor subject [13, 14]. However, despite being taught within the time and depth of content limitations of a minor subject, the mediation of broad and/or specific physics knowledge (details depending on the specific “non-physics” discipline) is important in these disciplines in the context of a knowledge foundation for subsequent studies in the major subject. For instance, in veterinary education, basic physics knowledge and comprehension is crucial to attending to biological or medical topics such as biomechanics (e.g., mechanics of musculoskeletal systems and their disorders and traumata, but also fluid dynamics of blood), diagnostic imaging technologies (e.g. magnetic resonance tomography and computer tomography), and radiation safety of X-rays. Usually, as in the case presented here, typical mandatory physics introductory courses for veterinary students consist of classic university teaching methods, such as lectures and practical laboratory courses. To complement, we designed a European boardgame-inspired educational escape room that combines thematic physics puzzles, optional puzzles, and a scoring system to foster student engagement and interdisciplinary learning. An implementation of this escape room was indented in the form of an additional elective physics course in the veterinary curriculum for first-year students. Although scoring systems are one of the most frequently implemented game elements [15, 16], the Eurogame-like concept for educational escape rooms encompasses more than just the pure victory point system. The concept of the *educational Eurogame Escape Room* includes:

- A defined teaching content or educational issues which can be very broad or specific (in this case fundamentals in physics);
- The mentioned victory point system as used in Eurogames;
- A strongly reduced interaction with the educational Game Master;
- Optional secondary puzzle (so-called *Eurogame puzzle* to earn additional victory points to compare the points with other groups of players), and therefore
- Individual or semi individual paths for players;
- Eurogame puzzles may contain atmospheric elements of board games such as chess, backgammon, or card games like skat (which are not necessary Eurogames, but important for a game-like association);

Combining escape room challenges with board-game-inspired mechanics offers promising and individual paths: students solve progressive physics puzzles with the option of additional, parallel tasks, earning optional victory points and fostering both cooperative (inside the teams) and competitive (compared with other teams by total victory points) motivation. A board game rulebook only interacts with the players in one direction, is neutral and nonjudgmental, which should also be attributed to the Game Master of the escape room for his passivity, who should therefore be predominantly absent and disappear like a game rulebook in the board game box. This allows open and unvalued communication between players without a hierarchical system during the game. To obtain a first impression and evaluation of the performance of the escape room concept, we conducted a pilot study with a limited number of veterinary students. In the evaluation, we indented to address the two research questions or, more precisely, objectives (i) the acceptance of the concept by the students and (ii) the increase or improvement in physics knowledge of the students, respectively.

**Fig. 1:**
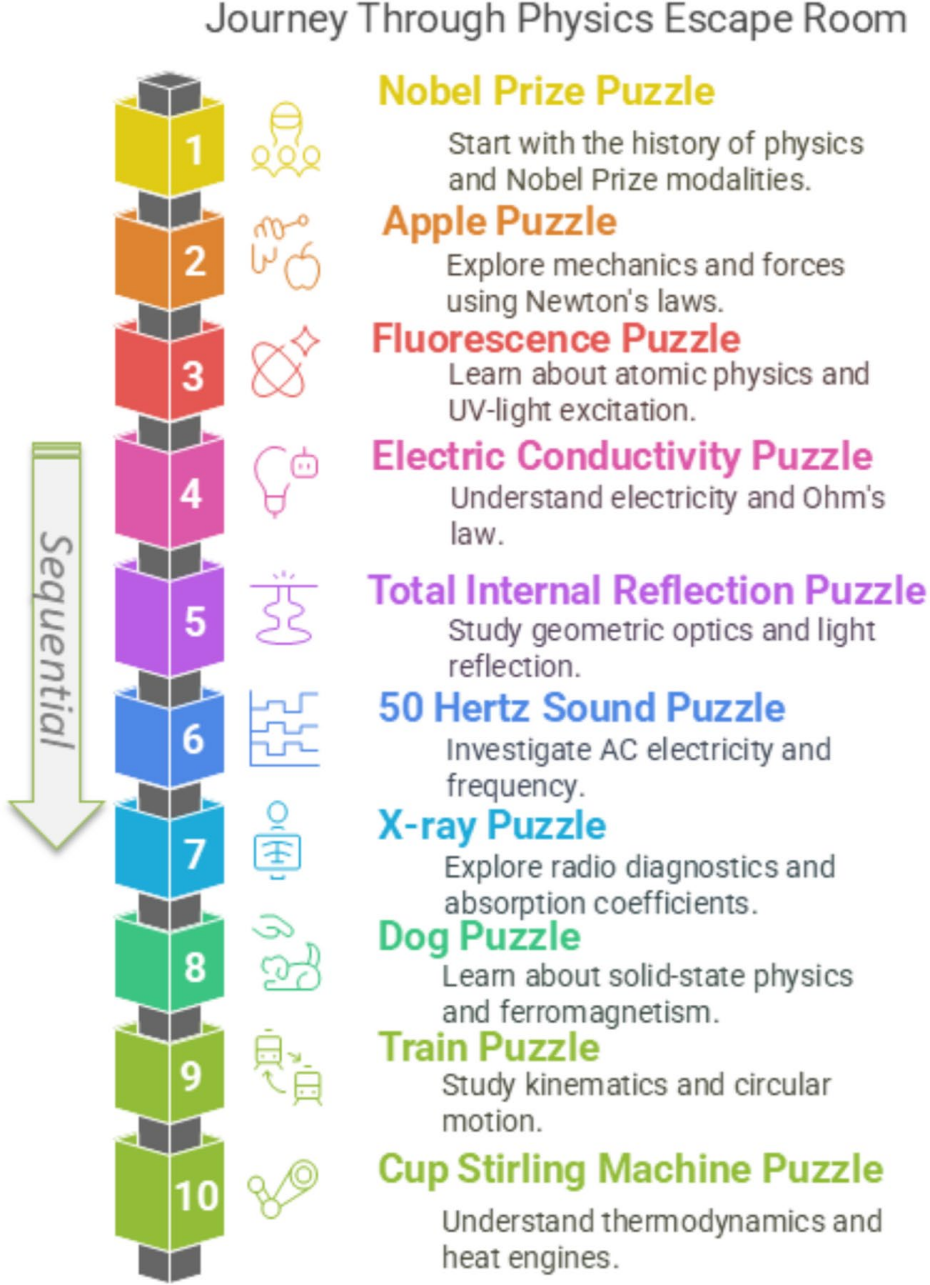
In this case, the educational escape room of physics contains 10 physics puzzles which must be solved in a sequential order and 16 optional puzzles (called Eurogame puzzles, unshown) with a total processing time of 2.5 hours. Eurogame puzzles can be solved in any arbitrary order

## Methods

**Fig. 2:**
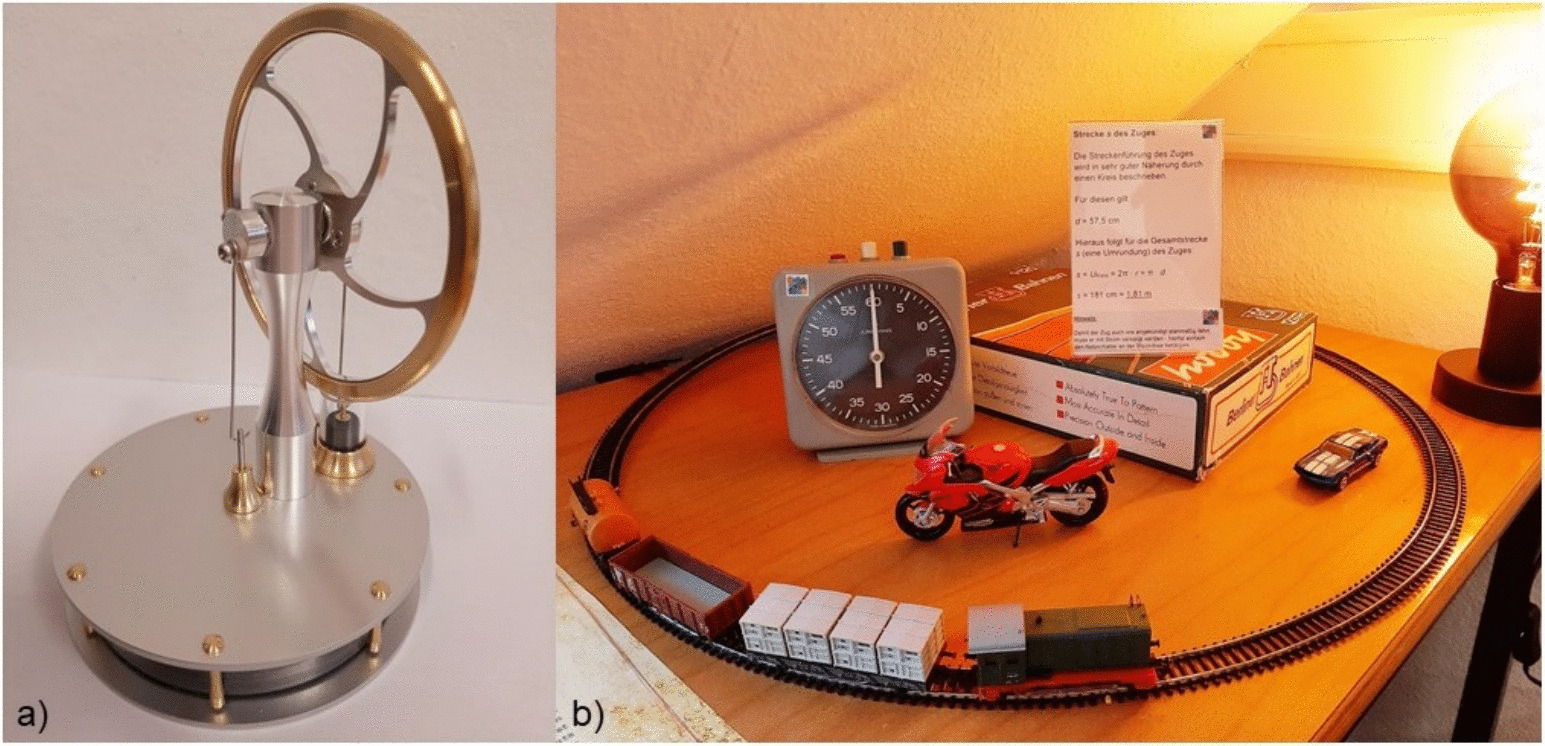
**a** Miniature Stirling engine which runs on several heat sources, e.g., cup of hot water, **b** train experiment to calculate the velocity of a moving model train on railroads by stopping a grey clock

**Fig. 3:**
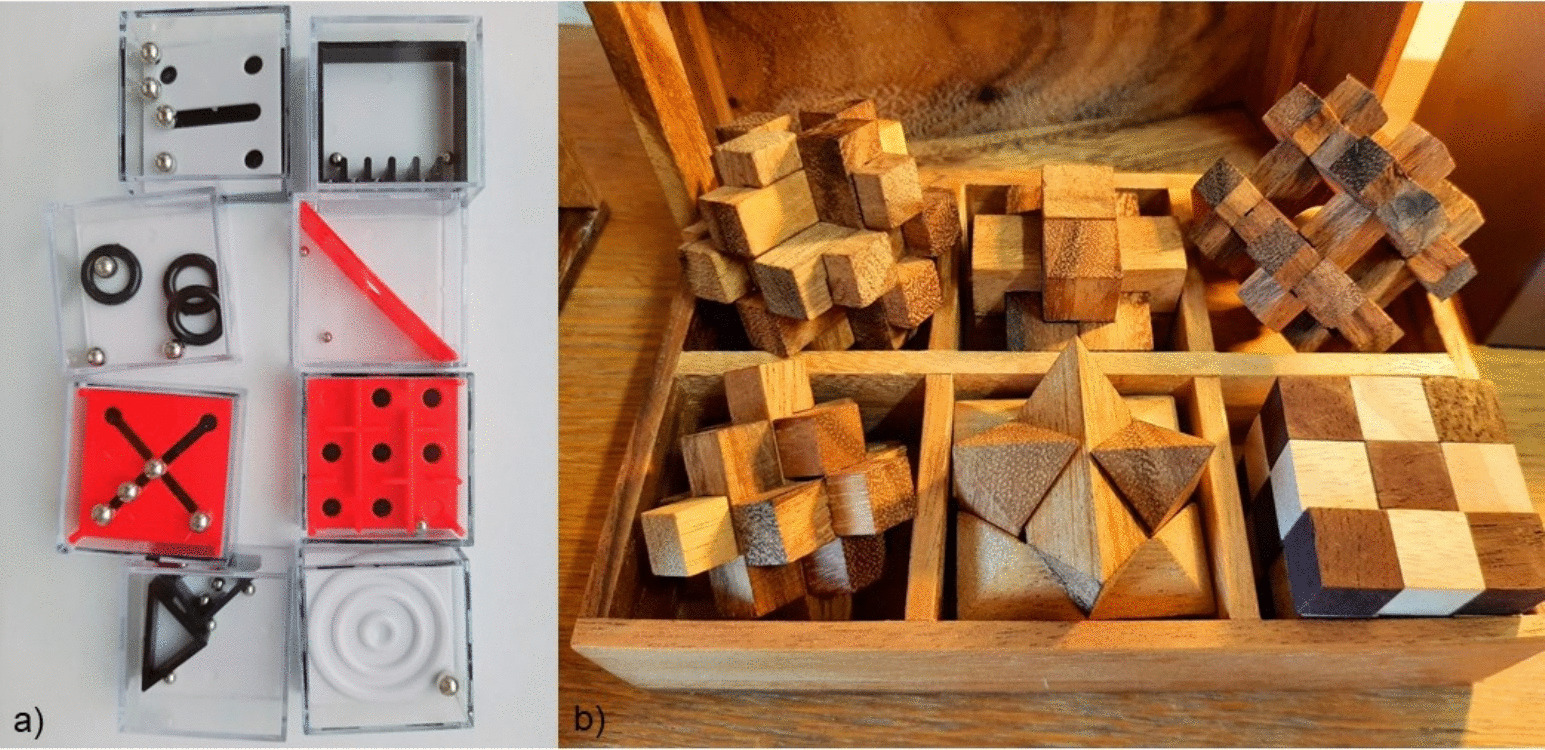
**a** Twenty-four dexterity cubes distributed in various boxes in the mirror room matching the modern environment (eight are shown) **b** six wood puzzles located in the rustic room called Schrödinger’s workroom

We developed a hybrid escape room in physics for first-year veterinary medicine students, who frequently have a low level of engagement with physics content. Students had a choice of subjects in the course catalog and could voluntarily choose the physics escape room. Approximately 85% of the students were female. The game was structured as a cooperative escape room using physical experiments, real puzzles, and printed materials. A game duration of 2.5 hours was scheduled per run. The participants, grouped into teams (optimum: team of 3 players), solved a linear sequence of physics puzzles, see Fig. 1 (for more details of the 10 physics puzzles and Eurogame puzzles, see supplementary material). Parts of the train puzzle and Stirling machine are shown in Fig. 2. The physics content covered most of the topics that have also been dealt with in a preceding/simultaneous mandatory basic physics lecture, e.g. thermodynamics, mechanics such as kinematics [17], electromagnetism, and radiation physics. Therefore, the topics are relevant to the exam and take into account constructive alignment [18]. Where applicable, it was also intended to embed the physics puzzles within a direct veterinary context to further increase the engagement of the students, since it is known in the literature that a combination of physics content with the speciality or, respectively, major subject of the students can be beneficial with regard to learning success and engagement of non-physics majors [14]. The escape room consisted of two rooms with a sequential lock system [19] of boxes supplemented by a light concept [20], a story line and historical text documents [21]: The students had to solve the physics puzzles to unlock the final challenge in the second room. This solving was mandatory to pass the course and the physics puzzles could be solved by the students by applying their knowledge acquired in the obligatory physics lecture. As an additional support, knowledge transfer sheets with short but precise repetitions for each core concept covered in the escape room physics puzzles were provided. However, to enhance the competitive motivation, the physics puzzles also resulted in victory points (the respective number of victory points per puzzle depended on the puzzle difficulty level) when solved, and assistance could be purchased from the Game Master (by telephone) in exchange for/loss of some points. In addition to the main puzzles, the students could tackle optional, open-ended side puzzles (so-called *Eurogame puzzles*) inspired by modern European board games, earning additional victory points. Two examples are shown in Fig. 3, it should be mentioned, we have included Eurogame puzzles with chess, backgammon and card game elements as well (see supplementary). In total, the victory point system, inspired by the mechanics of Eurogames, rewarded correct solutions (for both the physics and the optional puzzles), independent processing of the puzzles (especially with regard to the physics puzzles), as well as team-based and strategic problem solving. To assess knowledge gain and, thus, study objective (ii), students completed subject-specific knowledge transfer questions before and after the session anonymously. Each puzzle targeted a specific concept of physics and the prompts required qualitative reasoning and basic calculations. At the start of the study, each student completed a pretest (see supplementary material) to assess basic physics understanding. After participating in the escape room as a compulsory elective or voluntary course, respectively, the students completed a parallel posttest and a feedback questionnaire on their motivation (evaluation of objective (i)), the educational value of the activity, the impact of the Europuzzles and the victory point system. It should be noted that the combination of pre- and posttest questionnaire is an integral part of the educational escape room. The items will remain in place independent of there is a deliberate intention to conduct an evaluation. If a discussion of physics is induced by the first questionnaire afterwards, in combination with the teaching materials and questions answered directly by the puzzles, then this is only intentional and welcome. The detailed teaching methodology, process, and precise time schedule is divided into different phases. The aims and constraints implemented encompassed several key components and issues, which are outlined as follows:

- Phase 1: Introduction preparation room (6 minutes) On arrival, the students were welcomed into a designated preparation area located in the basement, which was equipped to store their clothing and backpacks for secure storage. Subsequently, they took their seats during Phases 1 and 2. The pedagogical framework and the operational mechanisms of the educational Eurogame escape room were briefly described. This involved a detailed explanation of how the escape room setting is utilized as an educational tool to enhance learning experiences by integrating problem-solving tasks and collaborative challenges that align with educational objectives. This was important because not all students have been familiar with the innovative format, highlighting that the escape room had been specially designed for veterinary students. Consequently, the complexity of the material aligned with the content of the lecture and the expectations inherent in the degree program. These intentions of the thoughtful comments served to reduce anxiety about physics [22] and put students at ease alleviating apprehensions related to physics, fostering a more relaxed atmosphere. In addition, it is imperative to recognize that the format is specifically tailored for first-semester students. Therefore, a prudent approach is essential to ensure its effectiveness. In the same room, followed:
- Phase 2: Questionnaire I for (ii), pretest knowledge gain (8 minutes) It was elucidated that the participants would be provided with an evaluation form designed to assess their level of knowledge. This instrument would function as a metric to quantify their advancement in the educational process. The formulation was precise and explicit, ensuring that the process excluded any form of grading involved. If they were unable to respond correctly to the majority of the questions, it was by design. The intent was to ensure that the solutions were not readily apparent from the lecture content. This strategy was employed to maintain a level of measurability within the context of evaluative assessment. The rationale behind the explanation was aimed at alleviating anxiety related to physics and mitigating any unforeseen elevated expectations. In order to maintain anonymity in responses, questions were addressed without revealing the identity of the respondents. Additionally, students were strategically seated at considerable distances from one another to prevent any potential interaction that could influence their responses.
- Phase 3: Introduction escape room (2 minutes) Following the administration of the questionnaire, the Game Master went with the players to the first room of the escape room located in the attic. Here, a countdown timer was configured to measure a duration of 2.5 hours. The telephone’s location was specified, and the participants were introduced to the general technical rules, as well as the documents necessary for engaging with the puzzles. It was elucidated that a primary objective was gaining access to the second locked room. Furthermore, an audio recording was referenced, which provided a concise overview of the rules and the thematic elements involved, specifically highlighting physics-related and Eurogame puzzles. This recording, which lasted approximately four minutes, served as a helpful additional introduction and repetition to the rulebook. Before the start of the audio briefing by the participants, the Game Master left the room but remained reachable by phone. This arrangement allowed the players to engage in communication without external disturbances or surveillance.
- Phase 4: Processing time (2.5 hours) About one hour after the beginning of the activities, a quick visit was conducted to ask the status of the players and whether they can cope with the concept. This feedback encompassed a short check of operational flow and a discussion to identify the presence of any technical difficulties that might have arisen, for example, jammed code boxes.
- Phase 5: Determination of victory points (3 Minutes) After Phase 4, the scoring in the room of Phase 4 had started. The Game Master was responsible for tallying the victory points accumulated from all solved physics puzzles and completed Europuzzles. It should be noted that in instances where assistance was sought for a physics puzzle, the victory points associated with that particular puzzle were subjected to a reduction, specifically by fifty percent. After that, the Game Master and the players returned to the basement room of Phase 1.
- Phase 6: Questionnaire II and III for points (ii) and (i) (12 minutes) Within the confines of the preparation room, the students were provided with two distinct questionnaires: one aimed at assessing their knowledge gain (posttest) to answer question (ii) and another designed to evaluate various aspects such as their motivation, specific characteristics of the Eurogame escape room like the Europuzzles and the victory point system to answer question (i). In this setting, once again, a methodical approach was used to strategically position the students at considerable distances from each other. This strategic placement was implemented with the specific intention of minimizing any potential interactions that could influence or alter individual responses to questionnaires.
- Phase 7: Final discussion (5 minutes) Students had the opportunity to provide verbal feedback on various aspects, including elements they appreciated, unexpected observations they encountered, and recommendations for potential enhancement or improvements. This fulfilled the dual objectives of effectively managing the game and expressing gratitude towards the students, while also illustrating a willingness to be receptive and transparent in communication. Subsequently, the individuals exited the room of Phase 1.

To contextualise the above structure of the phases, it is adapted from the classic Eurogame *Caylus* published by *Ystari* in 2005 [10]. The game is a classic worker placement game and is also divided into seven different phases. There is an action phase, in which the main action takes place, and administrative phases, similar to the educational Eurogame escape room as presented above.

## Results

**Fig. 4:**
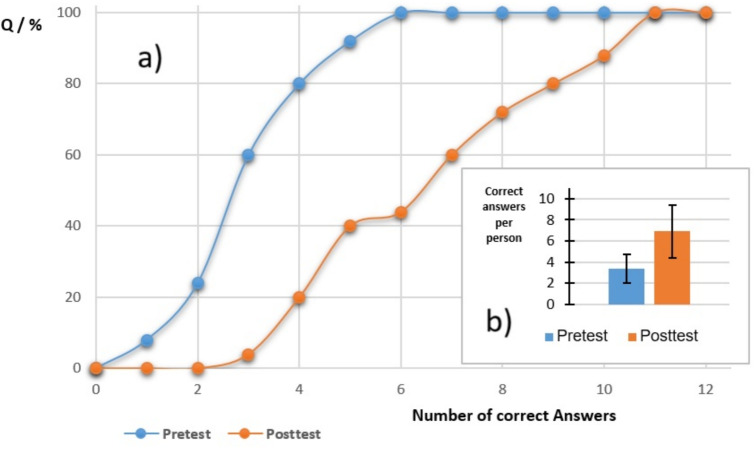
Statistical evaluation (correct answers) based on the total number of students ($N = 25$). **a** Pre- (blue) and posttest (orange) cumulative distribution *Q* of number of correct answers, **b** averaged numbers of correct answers, per person of pre- (blue) and posttest (orange) in comparison: arithmetic averages are 3.36 (pretest) and 6.92 (posttest), the error bars are the standard deviations of $s\approx \pm 1.35$ and $s\approx \pm 2.5$

In the limited pilot sessions, we had a total of $N=25$ students. These data have been extensively analyzed using statistical methods. In the following just the major results will be presented (for details, see supplementary materials). It should also be noted at this point that we only have the opportunity to collect these statistics from approximately 20–30 students once a year over a period of three months, which is related to the strict timetable and degree program of veterinary students in Germany. To address the first question (i) of acceptance, we asked anonymously *What is your opinion about the Eurogame puzzles and the associated victory point system?* In this case, 80% answered *good*, 20% *neutral* and nobody answered *bad*. To the question *How important are the Eurogame puzzles to you?*, they answered *important* (56%), *neutral* (36%) and *unimportant (8%)*. For question two (ii) of a knowledge increase, the participants showed a measurable improvement in the post-session knowledge transfer scores. Qualitative feedback indicated that the interactive format improved perceived relevance and enjoyment of physics content. Quantitative analysis showed a significant improvement in posttest scores compared to pretest results by various statistical methods [23–25], indicating effective knowledge transfer, see Fig. 4. The statistical distribution function was obtained by counting the incidence of the theoretical number of correct answers in the range 0 to 12 (number of questions of the various disciplines of physics; the detailed questions can be found in the supplementary material). The average number of correct answers per student has increased statistically significantly; see Welch’s t statistic in the supplementary data. Finally, the total number *T* of data points is given by the number of students *N* distributed on 12 various questions from physics disciplines: $T=300$. Key gains were observed in all disciplines, especially in disciplines that were directly addressed by multiple physics puzzles, such as mechanics and electromagnetism. Feedback indicated that most of the students found the narrative and authentic room setup highly motivating, with more than three-quarters rating the experience as more enjoyable and productive than traditional lectures or laboratory exercises. Students consistently reported that the victory point system, inspired by European board games, added an extra layer of motivation and entertainment. The system allowed them to choose side challenges based on their own interest or skill. In Phase 5, it was found that the individual choices of successfully completed Europuzzles varied greatly from group to group, and in Phase 7, the players of the groups explained and confirmed several times that this was due to the different individual preferences of the players, who each found different Europuzzles attractive or interesting. Most of the students reported increased engagement and found the combination of teamwork and individual tactical choice particularly attractive. The vast majority stated that the experience helped reduce apprehension towards physics.

## Discussion

It must be emphasized that the sample size $N = 25$ of the first pilot study presented in this work was relatively small, which, of course, limits the validity of the evaluation. To receive an extensive statistics, a long-time perspective over 5–6 years is required (especially in considering comparable setting of first-year students). This is planned for the further required and much more detailed evaluations of the designed concept. Nevertheless, the limited pilot study clearly shows the first positive results/trends with regard to the crucial objective (ii) the increase/improvement of physics knowledge of the participants. The integration of game mechanics, such as a victory point system and Eurogame puzzles, with domain-specific physics challenges created a motivational learning environment. The students actively applied the principles of physics to progress in the game, reinforcing their conceptual understanding. The veterinary context provided intrinsic motivation, particularly in clinically relevant topics such as imaging and dosimetry. Although the format requires preparation and facilitation, the reusability of materials and adaptability to other scientific disciplines make it a scalable model for interdisciplinary physics education. Based on the evaluation of the pilot study, the hybrid approach is indicated to be highly effective in improving motivation and learning in a group that typically does not identify with physics. The sequential main puzzles promoted teamwork and content focus, while optional side tasks enabled self-determined, interest-driven exploration and encouraged decision making and priority setting. According to self-determination theory, by incorporating the optional Europuzzles, individuals may experience an increase in their sense of autonomy, thus fostering a deeper engagement and commitment to the activity at hand and the primary task [26–29]. The choices available are reminiscent of strategic paths and choices in Eurogames where freedom of action plays a role. The element of board game rewards made the activity more game-like and approachable, bridging the gap between entertainment and rigorous academic content. In particular, the full integration of narrative and physical immersion was appreciated by the students, as was the structured provision of knowledge transfer sheets with concise explanations for each core concept. These served both as hints within the puzzles and as a learning reinforcement tool. Although the method was highly successful in this context of a limited pilot study, limitations can be that students may select easier side puzzles to maximize their points or to equalize point losses in the physics puzzles rather than challenge themselves with more challenging content areas and/or the attempt to solve as many of the physics puzzles as possible without assistance. Future implementations could improve this with tailored point weighting or by requiring more balanced completion across topics. In addition, the tests only examined the increase in factual knowledge. Experimental experiences, which are, in particular, the results of a practical physics course and the escape room, are not quantified and hard to extract [30, 31].

## Conclusion

Game-based learning can bridge the gap between abstract physics concepts and their practical applications in veterinary medicine. Our physics escape room demonstrates the potential of combining collaborative problem solving with strategic gameplay to promote active learning in non-physics student populations. Integrating board game principles such as Eurogame ideas into an educational escape room creates a flexible and motivating environment for interdisciplinary physics education. Our study demonstrates (i) a certain level of acceptance, and (ii) robust knowledge gains within the limited pilot session. This is supplemented by high student satisfaction, especially among students who are otherwise less engaged in physics, so far the feedback of personal discussions. Additionally, the players appreciated the optional offer of an analogue escape room in highly saturated digital times. This hybrid model, which combines structured core challenges with optional side quests and cooperative elements (within the group of players) and competitive elements (comparison of victory points on a scoring list presented by virtual group names), could be adapted for a variety of subjects and educational levels. Based on feedback, it seems to be a promising approach to mix an educational escape room with selected Eurogame concepts. Of course, improving the statistics and validity is a task for the coming years. However, the results are not expected to change significantly. A very interesting question concerns fundamental theories. It is well known that some European board games simply overwhelm players with game materials and cards. However, they are played in leisure time, are commercially viable, and are popular. This motivated the Eurogame puzzles used. These push the limits of cognitive load theory [32, 33] and offer a very interesting in-depth understanding of the human psyche that goes beyond classical theories and the didactics of physics.

## Supplementary Information

Additional file 1. The scope of the entire educational escape room is very extensive, with 10 physics puzzles + 16 Eurogames puzzles. For the sake of clarity, we have limited ourselves to the core content. The supplementary material contains two overviews of the selected puzzles (Physics puzzles and Eurogame puzzles), an extensive data analysis, and the questionnaire used

## Data Availability

No datasets were generated or analysed during the current study.
