# Supplementary material for: Eurogame escape room of physics in veterinary education: a pilot study
Source: BMC Vet Res. 2025 Dec 20;22:48. doi: 10.1186/s12917-025-05186-w (PMC12849234; doi:10.1186/s12917-025-05186-w)
Supplement: Supplementary file 1 — Additional file 1. The scope of the entire educational escape room is very extensive, with 10 physics puzzles + 16 Eurogames puzzles. For the sake of clarity, we have limited ourselves to the core content. The supplementary material contains two overviews of the selected puzzles (Physics puzzles and Eurogame puzzles), an extensive data analysis, and the questionnaire used [file 12917_2025_5186_MOESM1_ESM.pdf]

Statistical Evaluation 2
Physics Puzzles 10
Eurogame Puzzles 20
Questionnaire Knowledge Transfer 35

Supplementary Information: Statistical Evaluation, Physics Puzzles, Eurogames Puzzle, Questionnaire Increase in Knowledge

Eurogame Escape Room of Physics in Veterinary Education

Sascha Albert Bräuninger[1]*, Damian Alexander Motz[1, 2], Matthias Lüpke[1], Hermann Seifert[1]

[1]: University of Veterinary Medicine Hannover Foundation, Institute of General Radiology and Medical Physics, Bischofsholer Damm 15, Gebäude 102, 30173 Hannover, Germany.

[2]: current address: Leibniz University Hannover, Institute of Sanitary Engineering and Waste Management, Welfengarten 1, 30167 Hannover, Germany.

*: corresponding author: Sascha.Albert.Braeuninger@tiho-hannover.de

Statistical evaluation

To check and quantify the increase in knowledge of the students by completing the physics escape room anonymised pre- and posttests were performed. Both tests consisted of the same 12 questions, which considered the major physics sections and topics essential for veterinary medicine studies. The questions were single-choice tasks offering 4 potential answers (one correct answer, two false answers as well as the "I do not know" / neutral answer).

In total, 25 students completed the physics escape room in the first test run presented in this study. The students were separated into 8 groups of 2 to 4 persons each, in particular five groups of 3 persons, two groups of 4 persons and one group of 2 persons.

Statistical evaluation of the data

The obtained results of the pre- and posttests were statistically evaluated without further normalisation or modification as raw data. An evaluation approach based on the total number of students (N = 25) was applied.

The number of correct answers in the pre- and posttest of each student was counted and evaluated in comparison. The statistical distribution function was obtained by counting the incidence of potential / theoretical number of correct answers $\#_{\text{correct answers}}$ in the range 0 to 12. Figure S1a presents the distribution functions of the pre- and posttest results in direct comparison as cumulative distributions. The corresponding calculated statistical parameters are shown in Table S1 and, additionally, in Figure S1b the averaged numbers of correct answers (mean number of correct answers per person / student) of pre- and posttest are depicted.

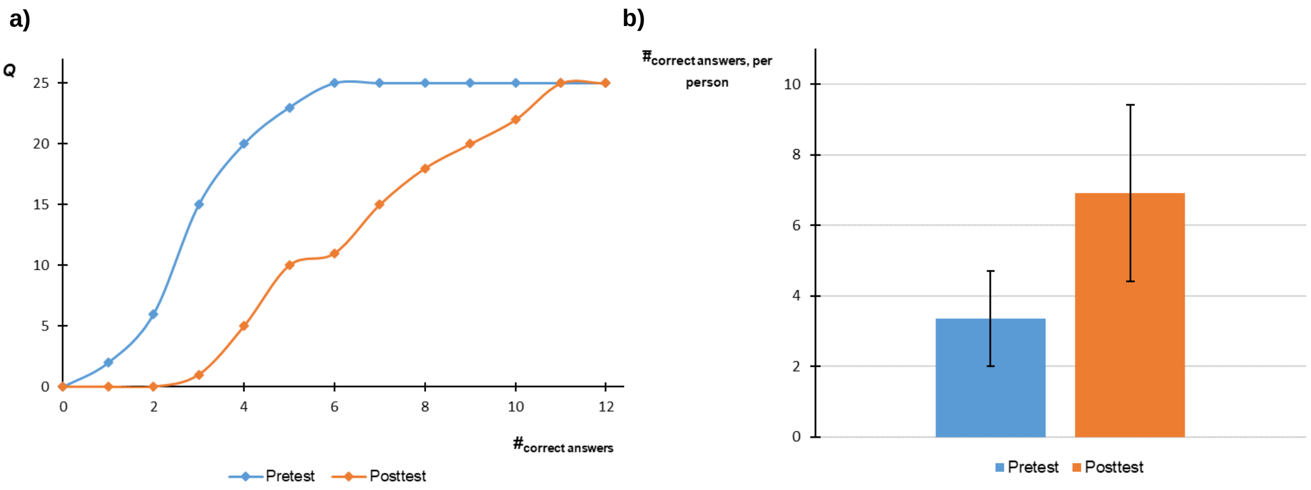

Figure S1: Statistical evaluation (correct answers) based on the total number of students (*N* = 25) – **a)** Pre- (blue) and posttest (orange) cumulative distribution *Q* of number of correct answers $\#_{\text{correct answers}}$, **b)** averaged numbers of correct answers $\#_{\text{correct answers, per person}}$ of pre- (blue) and posttest (orange) in comparison (the error bars are the standard deviations *s*).

Table S1: Determined statistical parameters of the evaluation (correct answers) based on the total numbers of students. The parameters of pre- and posttest are shown in comparison. The obtained results of a David test for normal (Gaussian) distribution are also presented.

**Statistical parameters**	**Pretest**	**Posttest**
Number of samples *N*	25 (= total number of students)	25 (= total number of students)
Arithmetic average $\#_{\text{correct answers}}$	3.36	6.92
Standard deviation[1] *s*	1.35	2.50
Variance s^2	1.82	6.24
Median *M*	3	7
Mode *D*	3	5
$\#_{\text{correct answers, min.}}$	1	3
$\#_{\text{correct answers, max.}}$	6	11
Range *R*	6 – 1 = 5	11 – 3 = 8
David test statistic *R* / *s* (David interval with *N* = 25, *P* = 90% [*α* = 0.10]: 3.45 - 4.53 [1])	3.45 < 3.70 < 4.53 → normally distributed	3.20 < 3.45 → slight variation from normal distribution

It is clearly visible (Figure S1a and S1b) that the quantity of persons achieving more correct answers as well as the average number of correct answers in the posttest and, therefore, after the students had completed the physics escape room, are distinctly higher compared to the

[1] Standard deviation *s* was calculated by $s_i = \sqrt{\frac{\sum(\#_i - \overline{\#}_i)^2}{N_i - 1}}$

pretest. Thus, this result provides first evidence that the required knowledge transfer was successful. Nevertheless, the high standard deviation especially of the posttest results and the overlap of the deviations of both test results are also noticeable.

To obtain statistically confirmed conclusions several statistical tests were applied. These tests were performed in *Microsoft Excel* both manually and step-wise based on the formulas (S1) – (S8) and, to check the manually calculated results, by using the *Excel* integrated *Analysis ToolPak*.

Since normal (Gaussian) distribution is one of the common assumptions for further statistical tests, a David test [1] was performed initially (see Table S1). With a confidence level of P = 90% the test statistics showed that the pretest data were normally distributed whereas the data of the posttest exhibited a slight variation from the normal distribution. This result had already been assumed regarding the differences in the values of the posttest arithmetic average, median and mode, because in case of a Gaussian distribution these three parameters are close to each other or, in case of an ideal Gaussian distribution, are equal. Therefore, in theory, a set of nonparametric statistical tests would deliver results that are more precise in the case of the posttest. Nonetheless, the posttest distribution was approximated by a normal distribution for the following statistical tests, since just the relatively small number of samples of the data set was assumed as the reason for the observed slight deviation[2]. However, it must be considered that this approximation / assumption can slightly limit the validity of the results.

Firstly, a two-tailed t-test (equal variances) [2] to compare the averages of the pre- and posttest results was performed. The test was based on the following hypotheses.

Null hypothesis: The averages of the pre- and posttest do not differ.

Alternative hypothesis: The averages of the pre- and posttest differ.

[2] The major deviating data point is at $\#_{\text{corr. answers}} = 6$. In the posttest data set, just one person achieved this number of correct answers (see Figure S1a).

The *t*-statistic was calculated with the formula (S1) [2].

$$t = \left| \frac{\overline{\#}_1 - \overline{\#}_2}{s_d} \right| * \sqrt{\frac{N_1 \cdot N_2}{N_1 + N_2}} \qquad \text{(S1)}$$

t: *t*-statistic, $\overline{\#}_i$: number of correct answers arithmetic average, s_d: weighted / pooled standard deviation, N_i: number of samples.

To apply (S1), s_d was calculated by using formula (S2) [2], beforehand.

$$s_d = \sqrt{\frac{(N_1 - 1) * s_1^2 + (N_2 - 1) * s_2^2}{N_1 + N_2 - 2}} \qquad \text{(S2)}$$

N_i: number of samples, s_i^2: variances.

The obtained t-test result is shown in formula (S3).

$$t = 6.267 > \ t_{crit.}(P = 99.9\%, d.o.f. = N_1 + \ N_2 - 2 = 48) = 3.505 \qquad \text{(S3)}$$

t: *t*-statistic (calculated by formula (S1) and (S2)), $t_{crit.}$: critical *t*-values (tabulated, see, e.g., [1] [2]), *P*: relative confidence level ($P = (1 - \alpha) * 100\%$, with α: significance level), *d.o.f.*: degree of freedom, N_i: number of samples.

The calculated *t*-statistic (6.267) is larger than the critical *t*-values at *P* = 95% (2.010), *P* = 99% (2.682) and *P* = 99.9% (3.505, see (S3)). Therefore, the null hypothesis must be rejected and, thus, the difference of the averages of correct answers of the pre- and posttest is statistically highly significant.

However, in detail, the validity of the standard two-tailed t-test depends on the homogeneity of variance. To survey the homoscedasticity an F-test [2] based on the following hypotheses was used.

Null hypothesis: The variances of the pre- and posttest do not differ (homogeneity of variance)

Alternative hypothesis: The variances of the pre- and posttest differ.

The calculation of the *f*-statistic was performed with the formula (S4) [2].

$$f = \frac{s^2_{higher\ variance}}{s^2_{lower\ variance}} \qquad \text{(S4)}$$

f: *f*-statistic, s_i^2: variances.

Formula (S5) presents the F-test result.

$$f = 3.424 > f_{crit.}(P = 99\%, d.o.f._1 = N_1 - 1 = 24, d.o.f._2 = N_2 - 1 = 24) = 2.659 \qquad \text{(S5)}$$

f: *f*-statistic (calculated by formula (S4)), $f_{crit.}$: critical *f*-values (tabulated, see, e.g., [1] [2]), *P*: relative confidence level ($P = (1 - \alpha) * 100\%$, with *α*: significance level), $d.o.f._i$: degrees of freedom, N_i: number of samples.

The determined *f*-statistic (3.424) was larger than the critical *f*-values at *P* = 95% (1.984) and *P* = 99% (2.659), which demonstrates that the variances differed statistically significantly.

Due to the inhomogeneity of variances and to overcome the limited validity of the standard t-test results, finally, a Welch's-t-test (modified two-tailed t-test with unequal variances) [3] was applied based on the same hypotheses as were used in the standard t-test.

The Welch's-*t*-statistic was calculated with the formula (S6) [3].

$$t = \frac{|\overline{\#}_1 - \overline{\#}_2|}{\sqrt{\frac{s_1^2}{N_1} + \frac{s_2^2}{N_2}}} \qquad \text{(S6)}$$

t: Welch's-*t*-statistic, $\overline{\#}_i$: number of correct answers arithmetic average, s_i^2: variances, N_i: number of samples.

The required estimation of the degree of freedom (*d.o.f.*) was performed using the Welch-Satterthwaite approximation (S7) [3].

$$d.o.f. = \frac{\left(\frac{s_1^2}{N_1} + \frac{s_2^2}{N_2}\right)^2}{\frac{s_1^4}{N_1^2 * (N_1 - 1)} + \frac{s_2^4}{N_2^2 * (N_2 - 1)}} \qquad \text{(S7)}$$

s_i^2: variances, N_i: number of samples.

The result of the Welch's test is shown in the formula (S8)

$$t = 6.267 > t_{crit.}(P = 99.9\%, d.o.f = 37) = 3.574 \qquad \text{(S8)}$$

t: Welch's-t-statistic (calculated by formula (S6)), $t_{crit.}$: critical t-values (tabulated, see, e.g., [1] [2]), P: relative confidence level ($P = (1 - \alpha) * 100\%$, with α: significance level), *d.o.f.*: degree of freedom (estimated by formula (S7)).

The obtained Welch's-t-statistic (6.267) is above the critical f-values at P = 95% (2.026), P = 99% (2.715) and P = 99.9% (3.574, see (S8)). Therefore, also the Welch's-t-test yield the statistically highly significant difference of the pre- and posttest averages of correct answers.

In total, the evaluation clearly demonstrated the increasing number of correct answers in the posttest and, thus, the general success of the developed physics escape room to increase the students' knowledge of physics. Additionally, the number of incorrect as well as neutral answers in the pre- and posttest of each student were also considered and evaluated the same way as the correct answers. Figure S2 shows the averaged numbers of wrong answers (mean number of wrong answers per person / student) (Figure S2a) and of neutral answers (mean number of neutral answers per person / student) (Figure S2b) of pre- and posttest in comparison. The corresponding calculated statistical parameters are presented in Table S2.

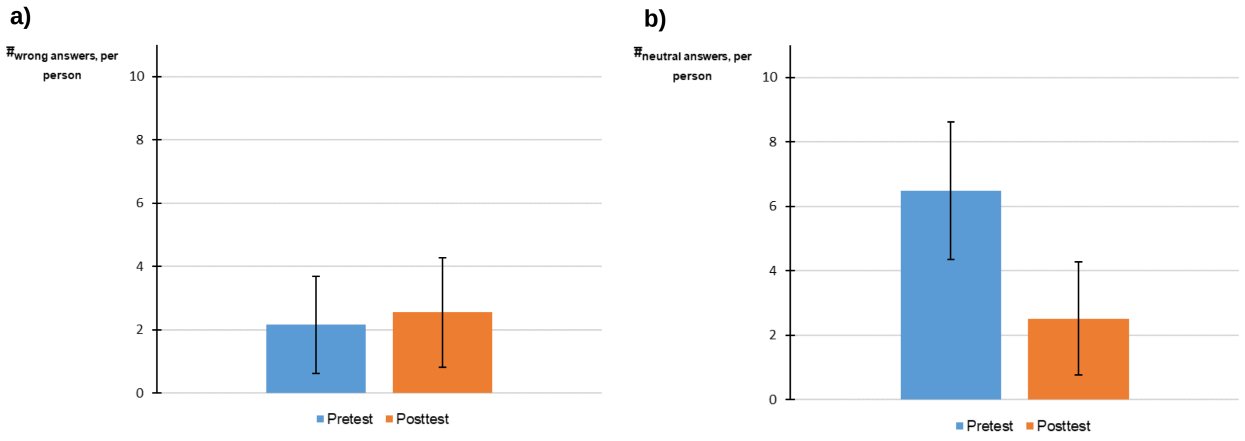

Figure S2: Statistical evaluation (wrong and neutral answers) based on the total number of students (N = 25) – Averaged numbers of **a)** wrong answers $\#_{\text{wrong answers, per person}}$ and **b)** neutral answers $\#_{\text{neutral answers, per person}}$ of pre- (blue) and posttest (orange) in comparison (the error bars are the standard deviations s).

Table S2: Determined statistical parameters of the evaluation (wrong and neutral answers) based on the total numbers of students. The parameters of the pre- and posttest are shown in comparison.

**Statistical parameters**	**Wrong answers Pretest**	**Wrong answers Posttest**	**Neutral answers Pretest**	**Neutral answers Posttest**
Number of samples N	25 (= total number of students)	25 (= total number of students)	25 (= total number of students)	25 (= total number of students)
Arithmetic average $\#_{answers}$	2.16	2.56	6.48	2.52
Standard deviation s	1.55	1.73	2.14	1.76
Median M	2	2	6	2
Mode D	2	3	5	5
$\#_{answers, min.}$	0	0	2	0
$\#_{answers, max.}$	5	6	11	5
Range R	5 – 0 = 5	6 – 0 = 6	11 – 2 = 9	5 – 0 = 5

The low values of wrong answers, both in the pre- and posttest, are demonstrative. Furthermore, a distinct decrease in the number of neutral answers in the posttest compared to the pretest is visible. These observations were confirmed by statistical tests analogous to the correct answer evaluation. Thus, e. g., the Welch's-t-test showed that there was no statistically significant difference between the averaged number of wrong answers of the pre- and posttest ($t = 0.861 < t_{crit.}$ (P = 95%, *d.o.f.* = 47) = 2.012). In contrast, regarding the neutral answers the difference was statistically highly significant ($t = 7.142 > t_{crit.}$ (P = 99.9%, *d.o.f.* = 46) = 3.515). This additional consideration of the wrong as well as neutral answers further confirmed the conclusions of the correct answer evaluation described above. The number of neutral answers decreased in the posttest. Since the averaged number of wrong answers remained nearly constant, the majority of the answers shifted from neutral to the correct answers. This proved the mentioned success of the physics escape room in increasing the knowledge of physics of the students. Furthermore, the evaluation of the wrong and neutral answers also demonstrated that most of the students avoided guessing correct answers in the questions (see relatively low averaged numbers of wrong answers in pre- and posttest and high numbers of neutral answers in the pretest), which was crucial for the statistical evaluation in general. On top of this, the rate of misconceptions during the knowledge transfer of the escape room was low (see low averaged numbers of wrong answers in the pre- and posttest), which was essential from the didactic perspective.

References

[1] W. Gottwald, Statistik für Anwender - Die Praxis der instrumentellen Analytik, U. Gruber and W. Klein, Eds., Weinheim, Berlin, New York, Chichester, Brisbane, Singapore, Toronto: Wiley-VCH, 2000.

[2] W. Funk, V. Dammann and G. Donnevert, Qualitätssicherung in der Analytischen Chemie, Weinheim: VCH, 1992.

[3] NIST/SEMATECH, "NIST/SEMATECH e-Handbook of Statistical Methods," NIST/SEMATECH, [Online]. Available: http://www.itl.nist.gov/div898/handbook/. [Accessed 19th February 2024].

This document contains images and details of the Physics Puzzles not presented in the main paper. We present a picture supplemented by a brief explanation. Of course, this can only be an outline. The train experiment is linked in the main text as well.

Nobel Prize: History of Physics

History of physics: The aim is to allocate data from Nobel Prize winners based on slips of paper that are hung next to them. The players should recognize what the Nobel Prize is for and be able to roughly classify the times. For example, it is important to realize that Albert Einstein did not receive the Nobel Prize in Physics for the theory of relativity.

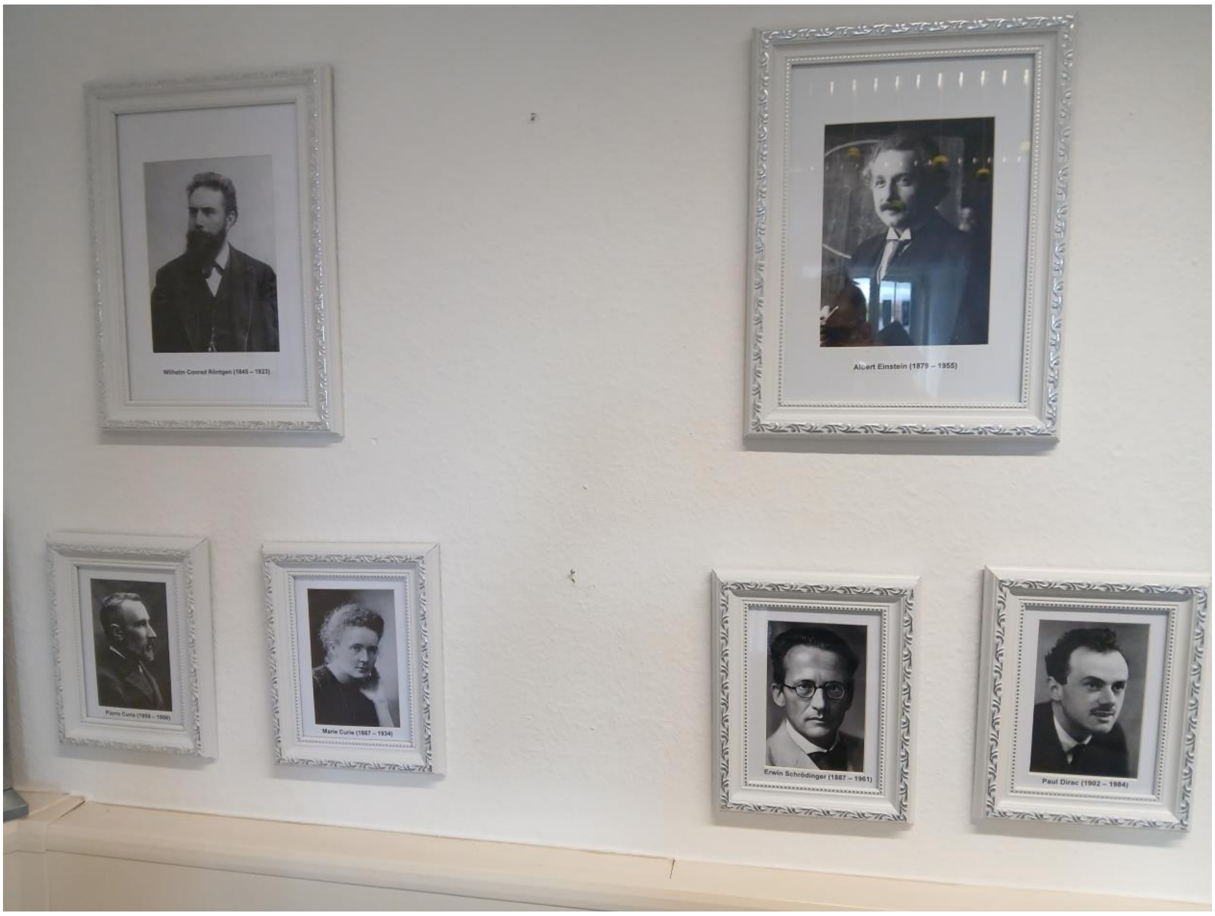

Apple puzzle: **Forces and Newton’s Axioms**

The aim is to calculate the code for the next box using the weight of the apple. This is about forces and the laws of classical mechanics and Newton's axioms. The apple is prepared the weights.

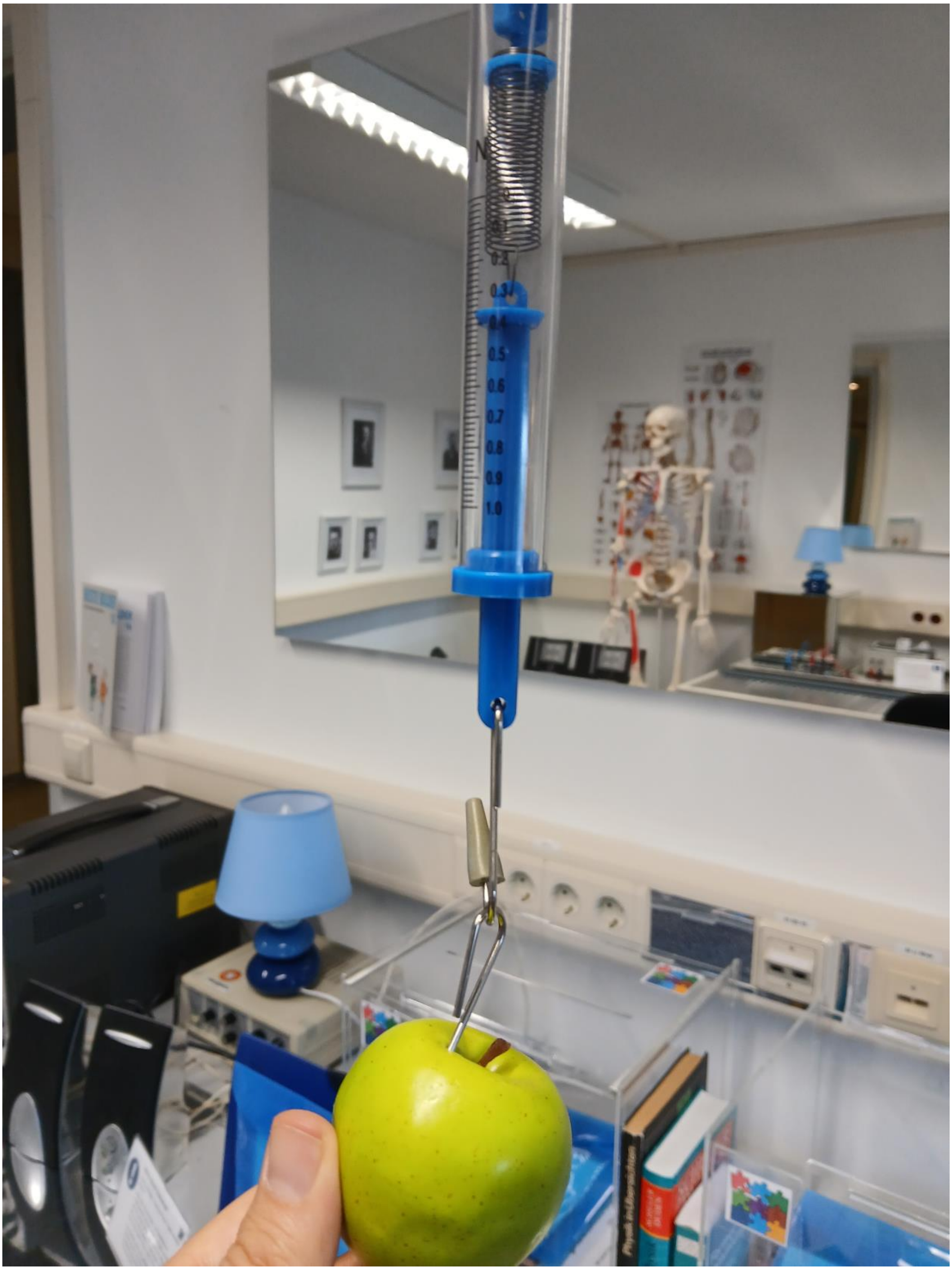

Electric Conductivity: DC Electricity

The players have to determine the conductivity of different materials, for example a bone (important in veterinary medicine), a metal and a mineral. This involves the use of a voltmeter.

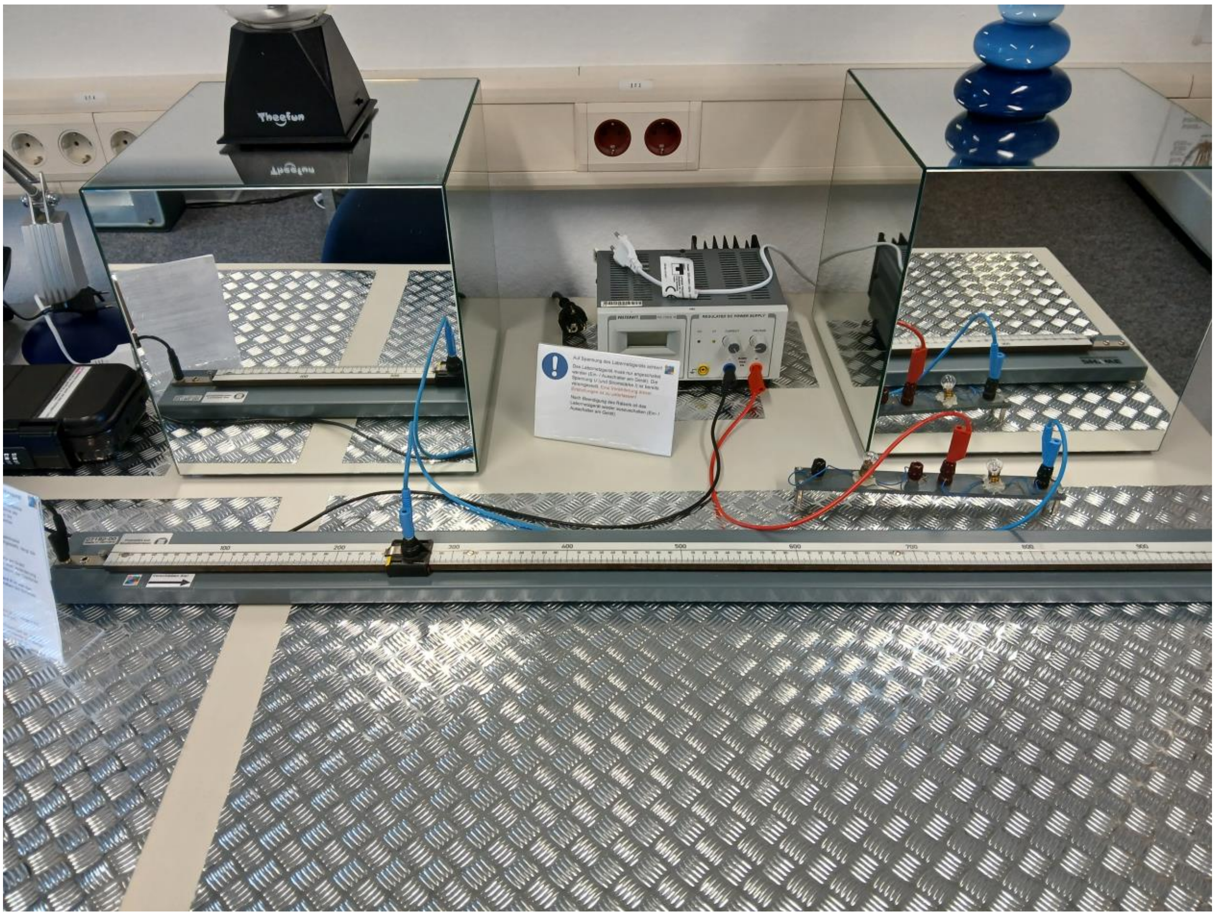

Fluorescence: Atomic Physics

The players have to put together a puzzle consisting of an image that only becomes visible under UV radiation. Here the students should learn that they must protect themselves with glasses, i.e. pay attention to safety aspects, and learn the basics about fluorescence (atomic physics, molecular excitation).

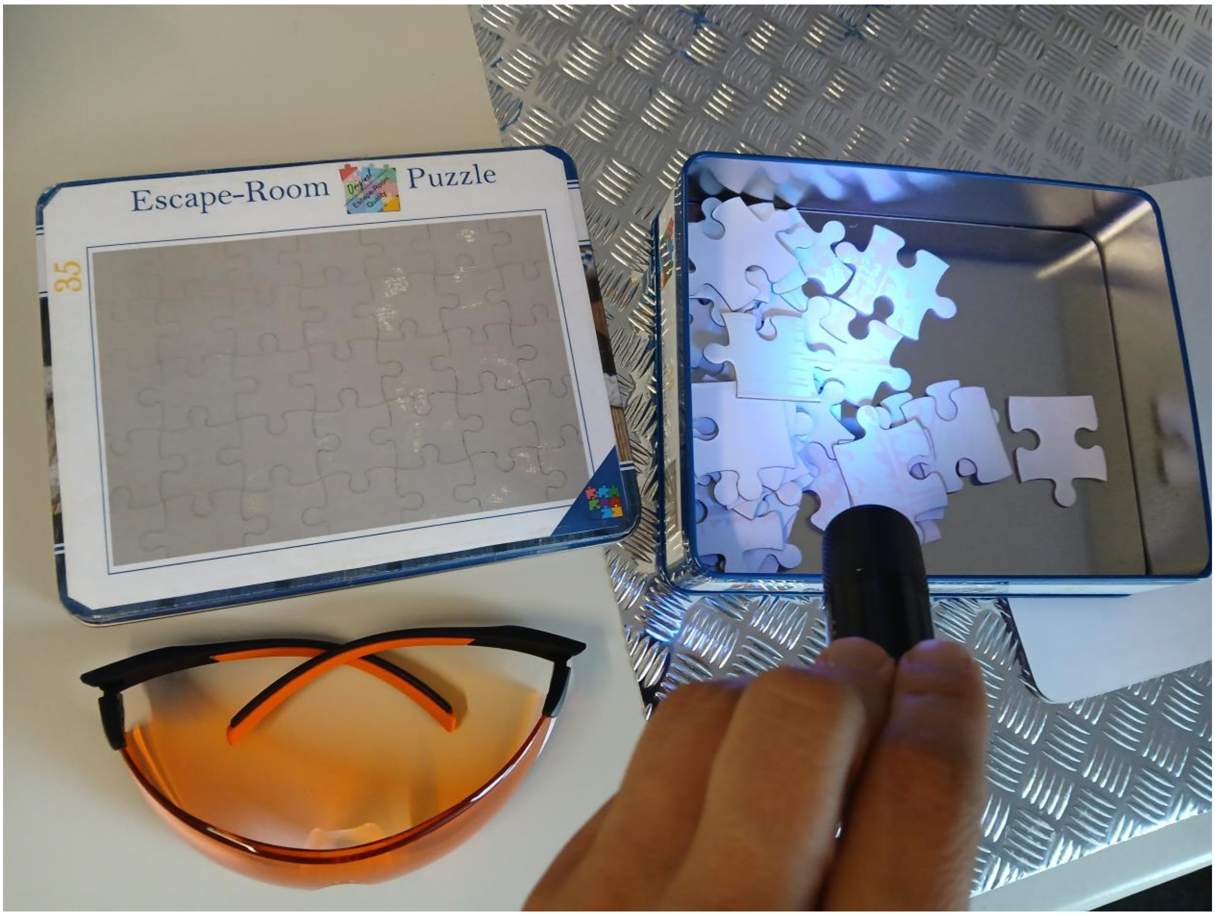

50 Hz Sound Experiment: AC Electricity

The students put a sound experiment into operation. The alternating current frequency is translated into sound using loudspeakers. The players are asked to read the 50 Hz on the scale.

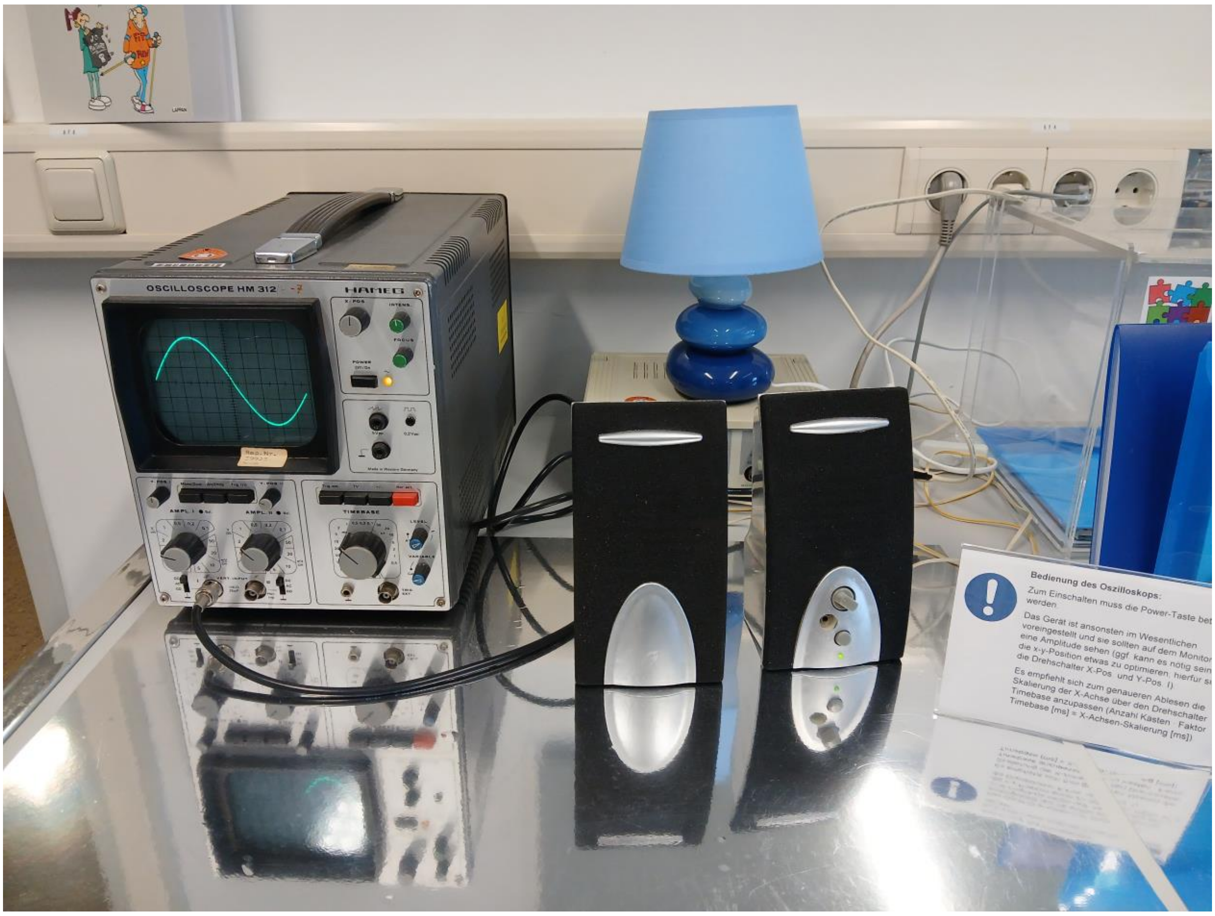

Total Internal Reflection: Optics

The players receive a numerical code for the next box based on the angle of the total reflection. The entire puzzle is mounted on the wall by magnets so that the players can arrange the elements themselves.

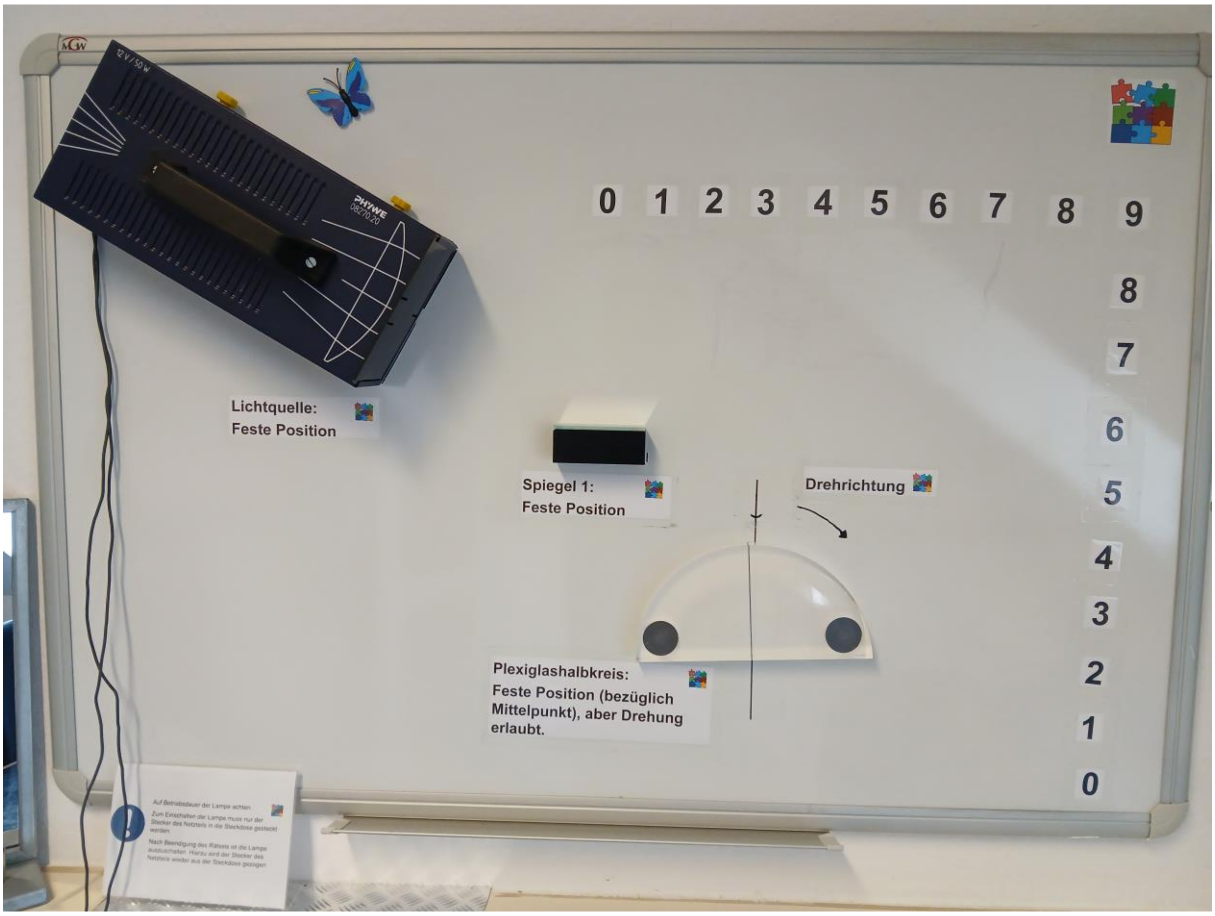

X-ray: Ionizing Radiation and Dosimetry

The players have to deduce the absorption coefficient of the X-rays from the brightness of the squares, which gives the players a numerical code. The puzzle is intended to arouse curiosity about the lectures on physical radiology in the higher semesters.

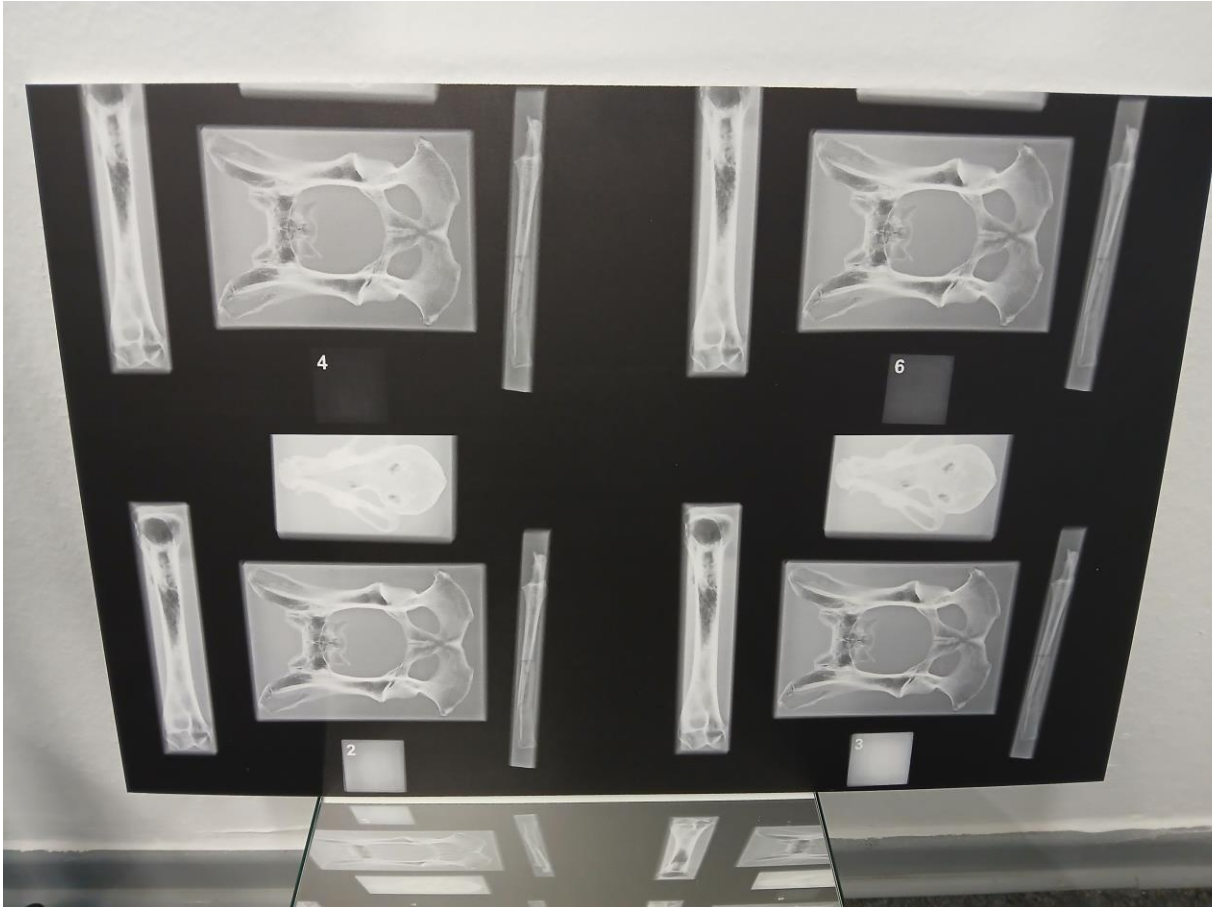

Stirling Engine: Thermodynamics

Miniature Stirling engine which runs on several heat sources, e.g., on a cup of hot water. In this case, hot water is hidden and the Stirling engine is the final puzzle and presents a puzzle to learn the basic knowledge of heat engines and thermodynamics.

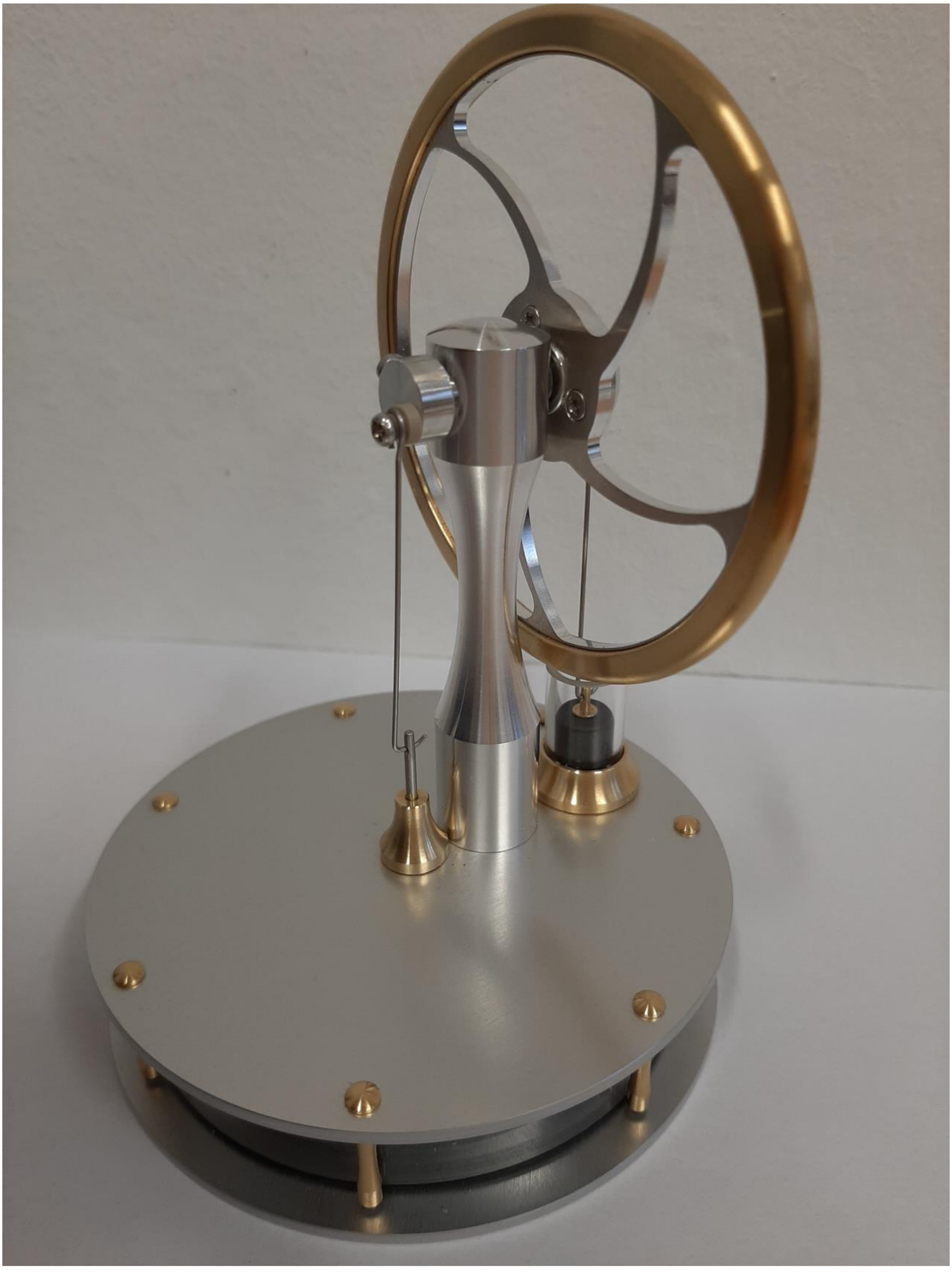

Dog Puzzle: Magnetism and Solid State Physics

Dog which consists of tetris-like blocks containing magnets inside to stabilize the construction of this puzzle. The separated blocks having stickers with the added numbers and symbols to achieve equations after puzzling the dog. It is important to consider the symmetry of the puzzle pieces. This yields the code for the next box. This puzzle has a high gamification level.

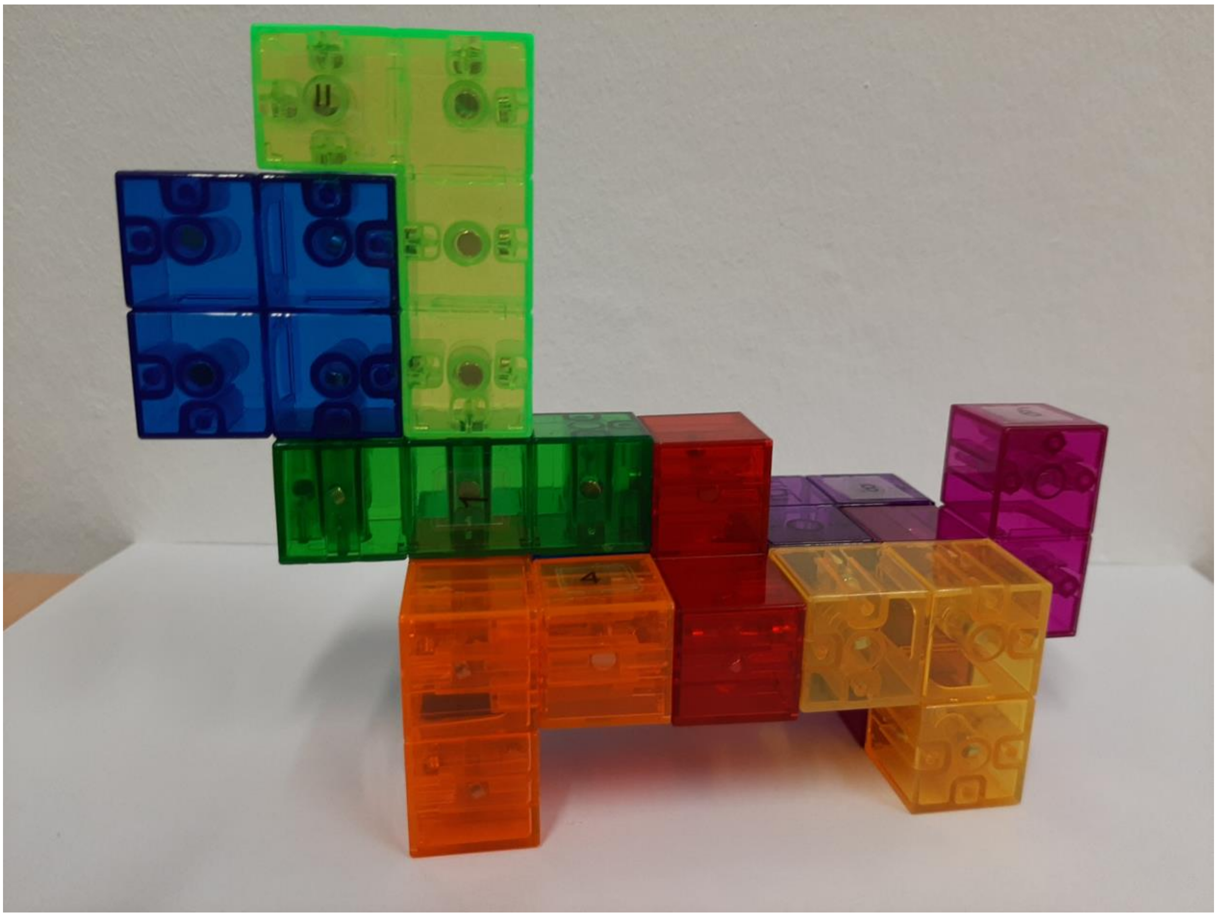

Train Experiment: Velocity and Kinematics

The train experiment is constructed to calculate the velocity of a moving model train on railroads by stopping a grey clock. The team of students calculate the velocity, the first two digits are the important numbers of the code for the next box.

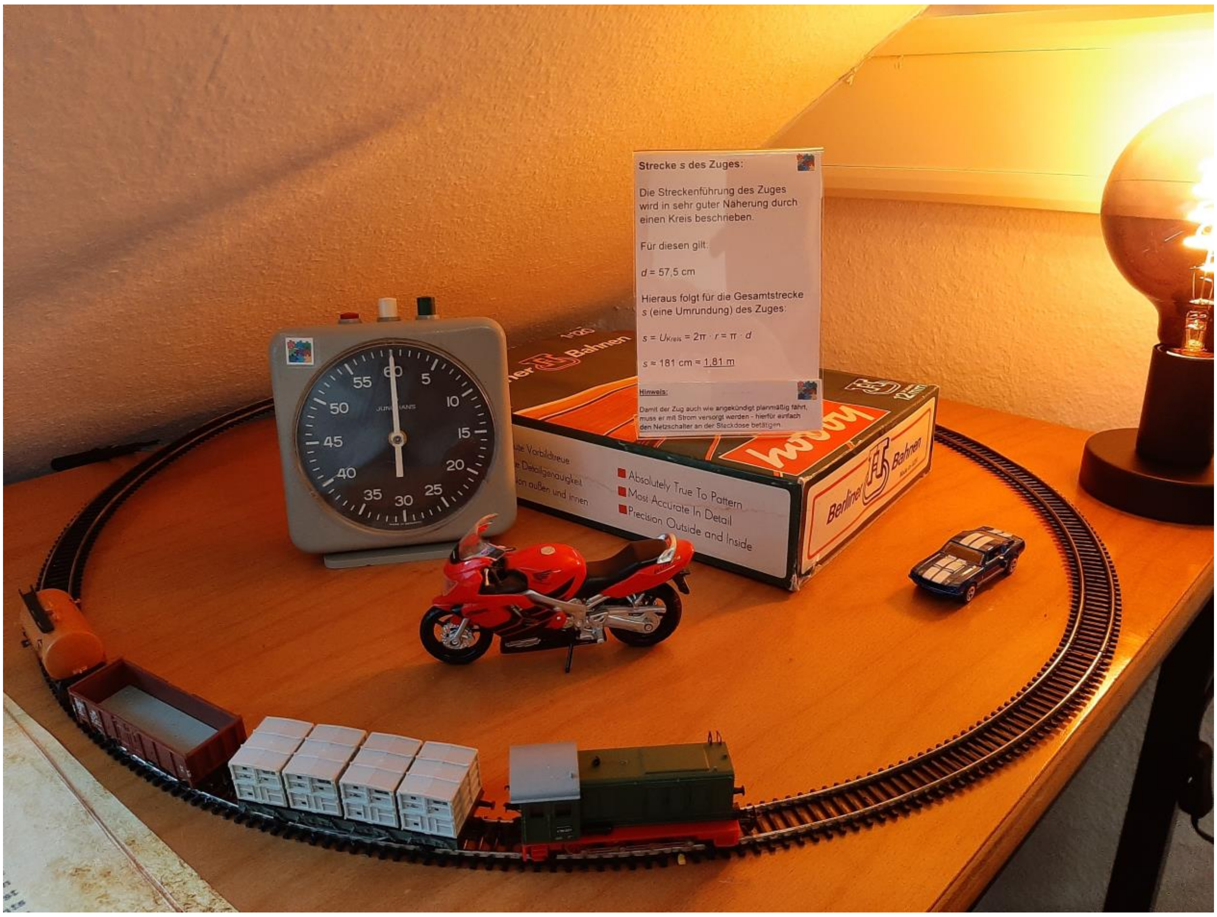

This document contains images and details of selected Eurogame puzzle. We present a picture supplemented by a brief explanation. Of course, this can only be an outline. Furthermore, some puzzles or parts are protected by copyright, as commercial solutions have been used. We therefore only show selected elements. Furthermore, this document is not intended as a solution book for students, which is why we have omitted too many details (e.g. solutions). It should be noted, the Eurogame puzzles are not always strongly connected to physics. The puzzles have motivating or even cosmetic character in terms of physics didactics. Some puzzles contains questions of chemistry, which is an important subject for students of veterinary medicine.

Backgammon

Here, checkers are stacked on a half-sided backgammon game. With a UV lamp (from the UV puzzle, see physics puzzle) you can make the letters in front of the stacked checkers recognizable (see photo, left bottom letters are lighting). The height of the checkers determines the order of the letters. The solution word is the name of an Indian physicist. The puzzle is difficult because you have to make the connection to the UV puzzle, which is in the other room. The letters also have to be put in order. The existing puzzles are listed in the rulebook, so you know that a Eurogame puzzle is the backgammon game. This puzzle is homemade.

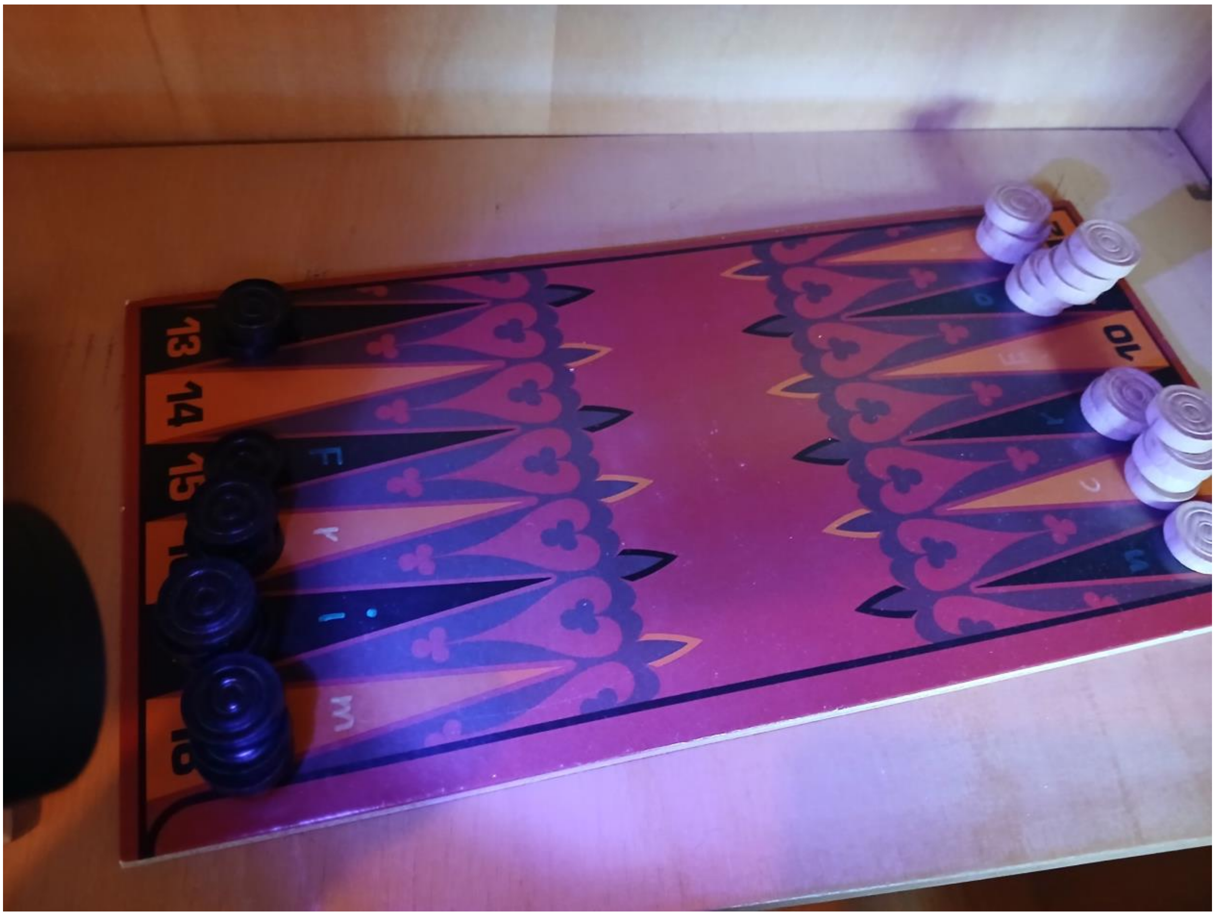

Biochemistry

The biochemistry puzzle contains representations of molecules printed on transparent film. These must be attached correctly with magnets. Reaction arrows are shown on the board. There is a pharmacy book in the second room in which you can look up the solution. It is important to look up iodine and make the connection to the thyroid gland. This is about looking things up in a book, skills that are no longer trivial in the digital age. The book is an old one. The solution is not shown below. Additionally, at the top right you can see the clock, a small timer with which the time of the escape room (processing time) was stopped. The puzzle is of medium difficulty and homemade.

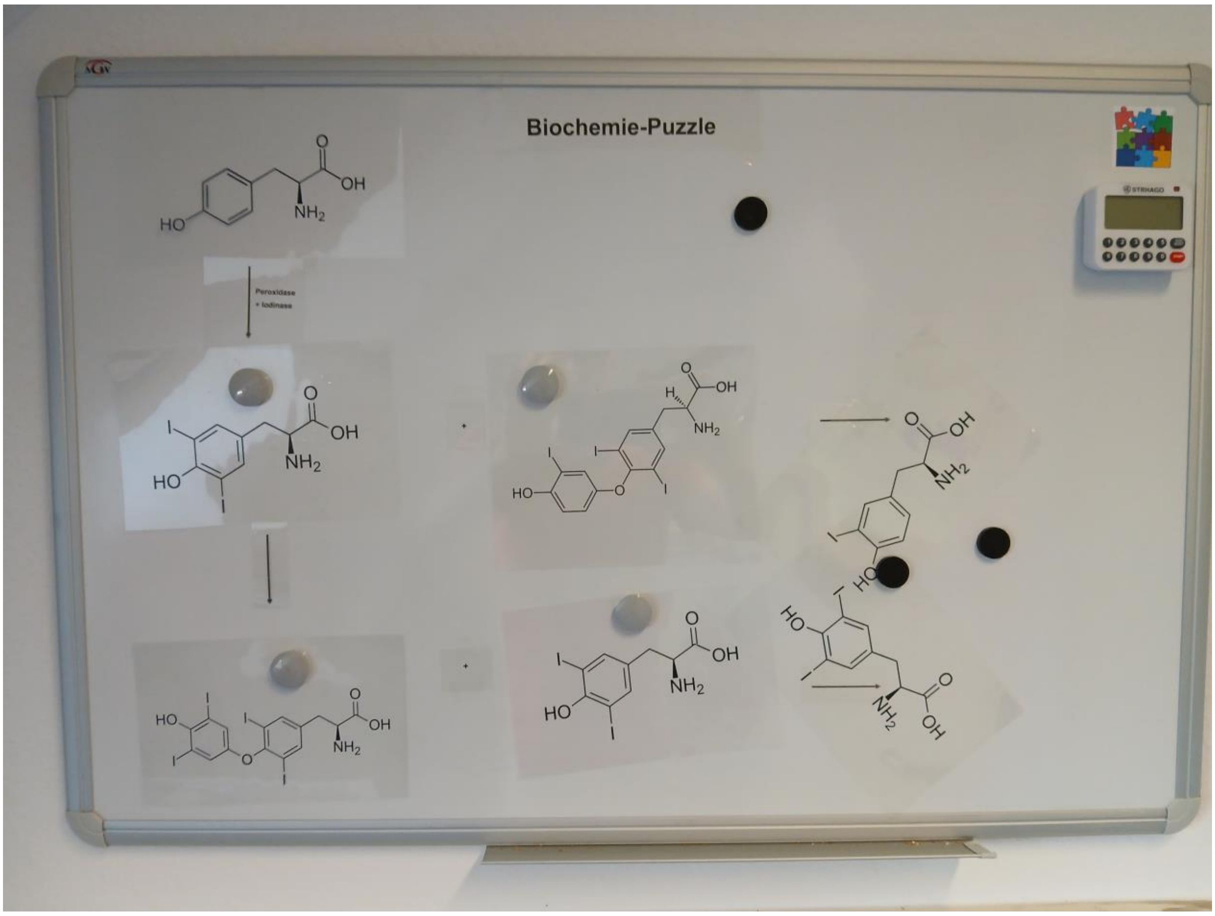

Domino

Dominoes are in a jar. The players have to use the UV light to make letters recognisable. The dominoes must then be sorted (all with letters) and arranged. They form the name of a physicist. The puzzle is difficult because you have to make the connection to the UV puzzle of physics. The puzzle is homemade.

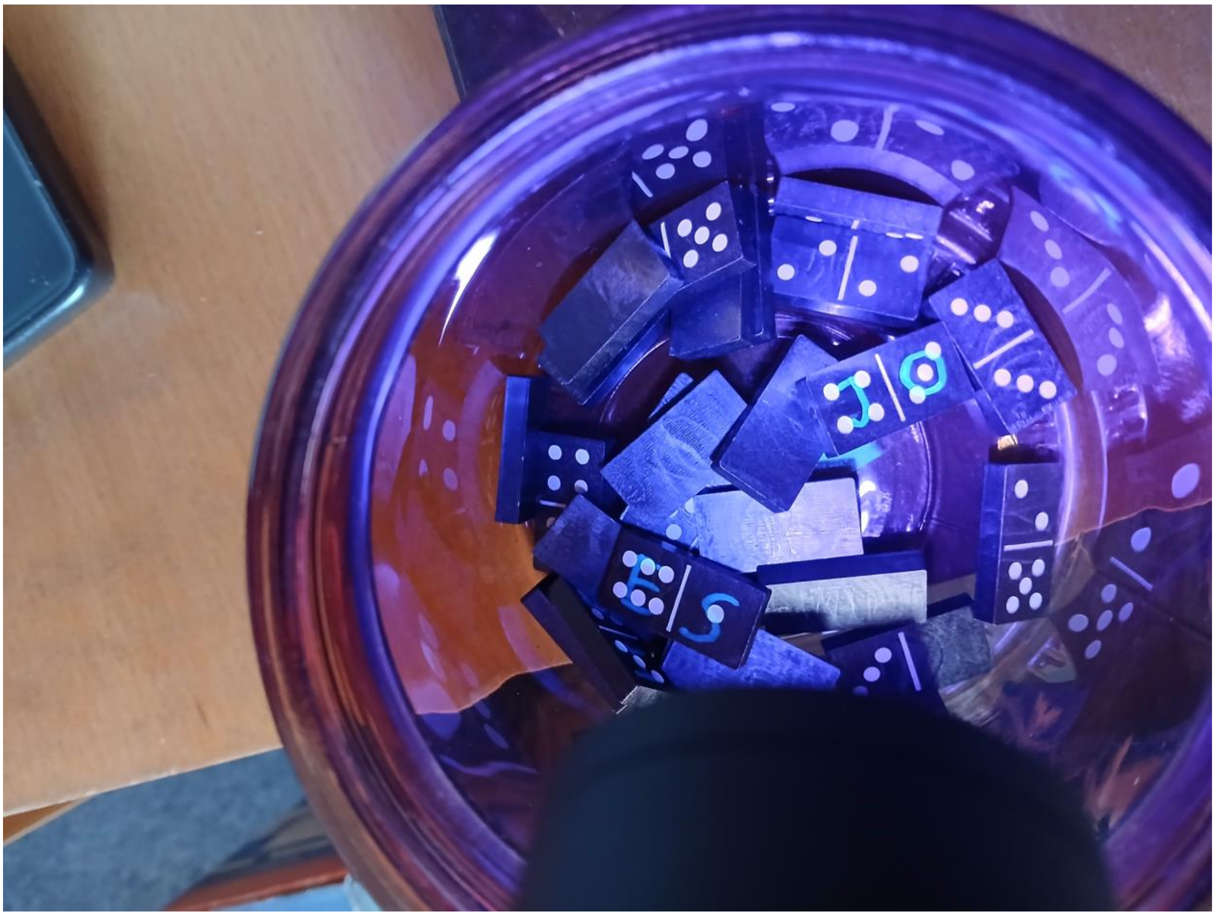

Einstein Look

The Einstein lock is a commercial solution from the *Moses* publishing house (*Professor Puzzle*). Tetris-like stones have to be moved with a small key to separate the lock from the cup. The puzzle is moderately difficult and trains spatial imagination and the ability to concentrate.

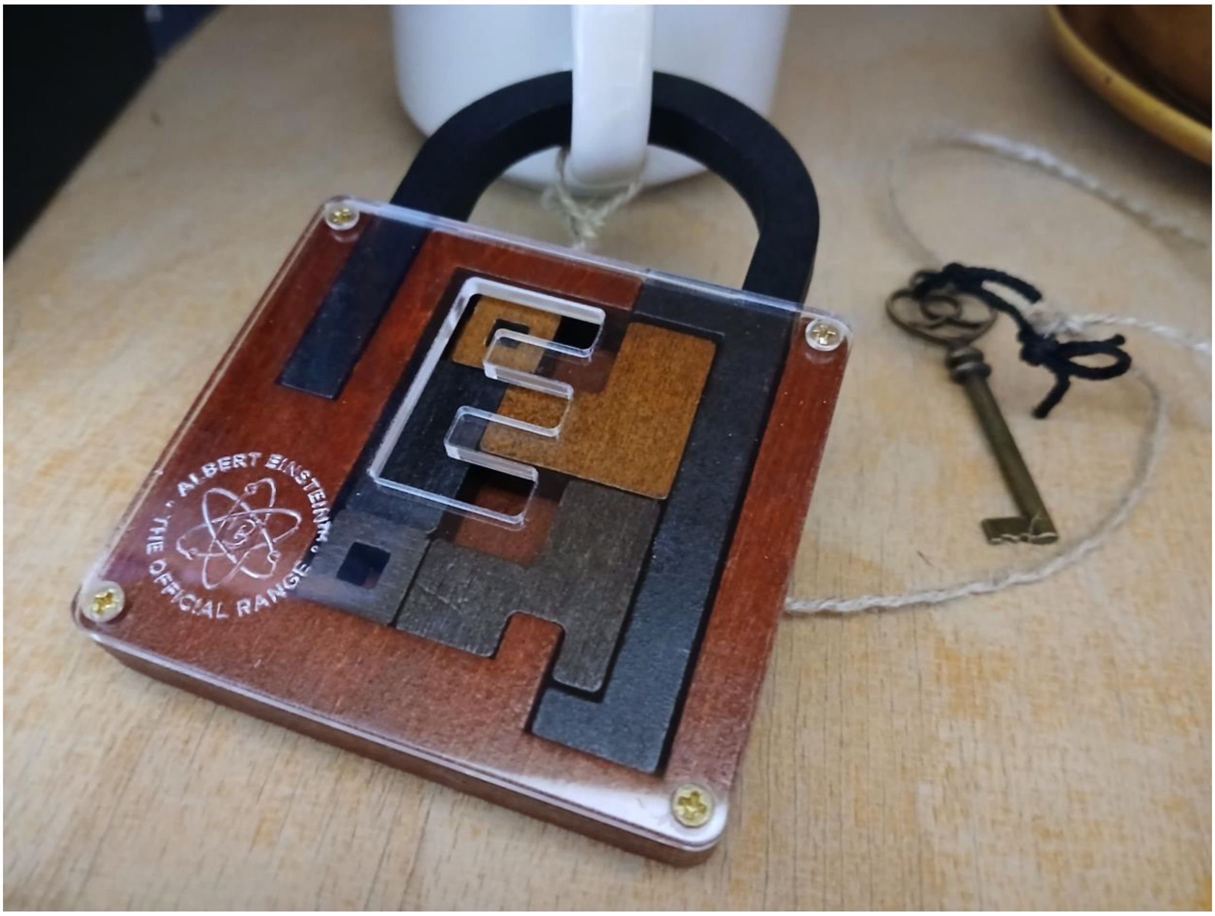

Bottle Safe

Another Eurogame puzzle is a classic bottle puzzle. It is a bottle safe in which a glass bottle has been found. You have to free it by arranging the wooden elements and ropes. Under the bottle is the name of a physicist, and the e-game master records the points when the safe has been successfully opened. Motor skills and spatial awareness are trained here, the puzzle is moderately difficult. It is a commercial solution bought from the company *LOGOPLAY* (bottle not included).

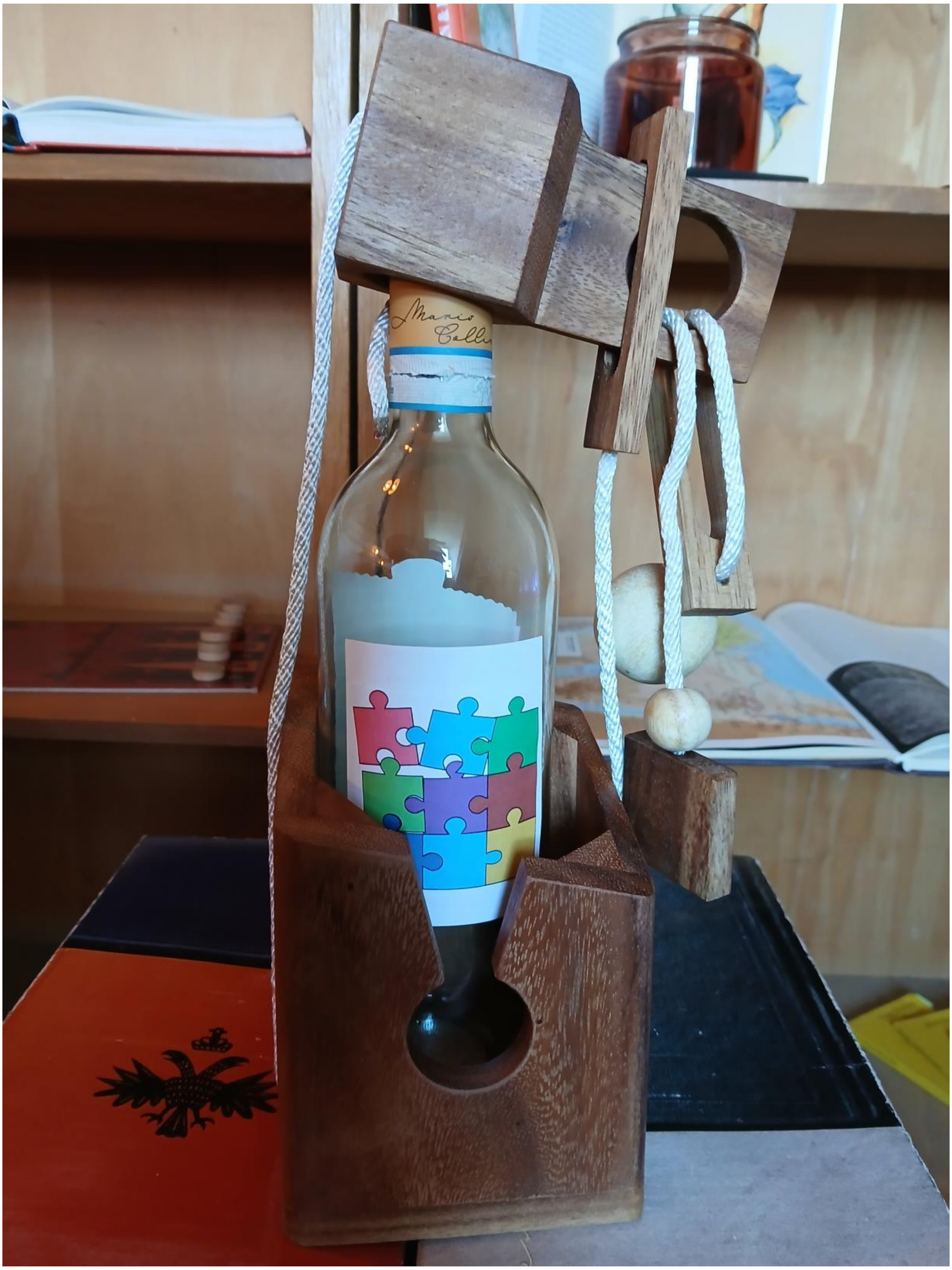

Card Game

There are small letters on playing cards that are in a small box (see front). You have to pick out the playing cards with the letters and arrange them in the order of a picture on the wall, on which there are also playing cards of the same type. The puzzle is of medium difficulty, the cards are commercially purchased, otherwise the puzzle is homemade. The result is the name of a physicist.

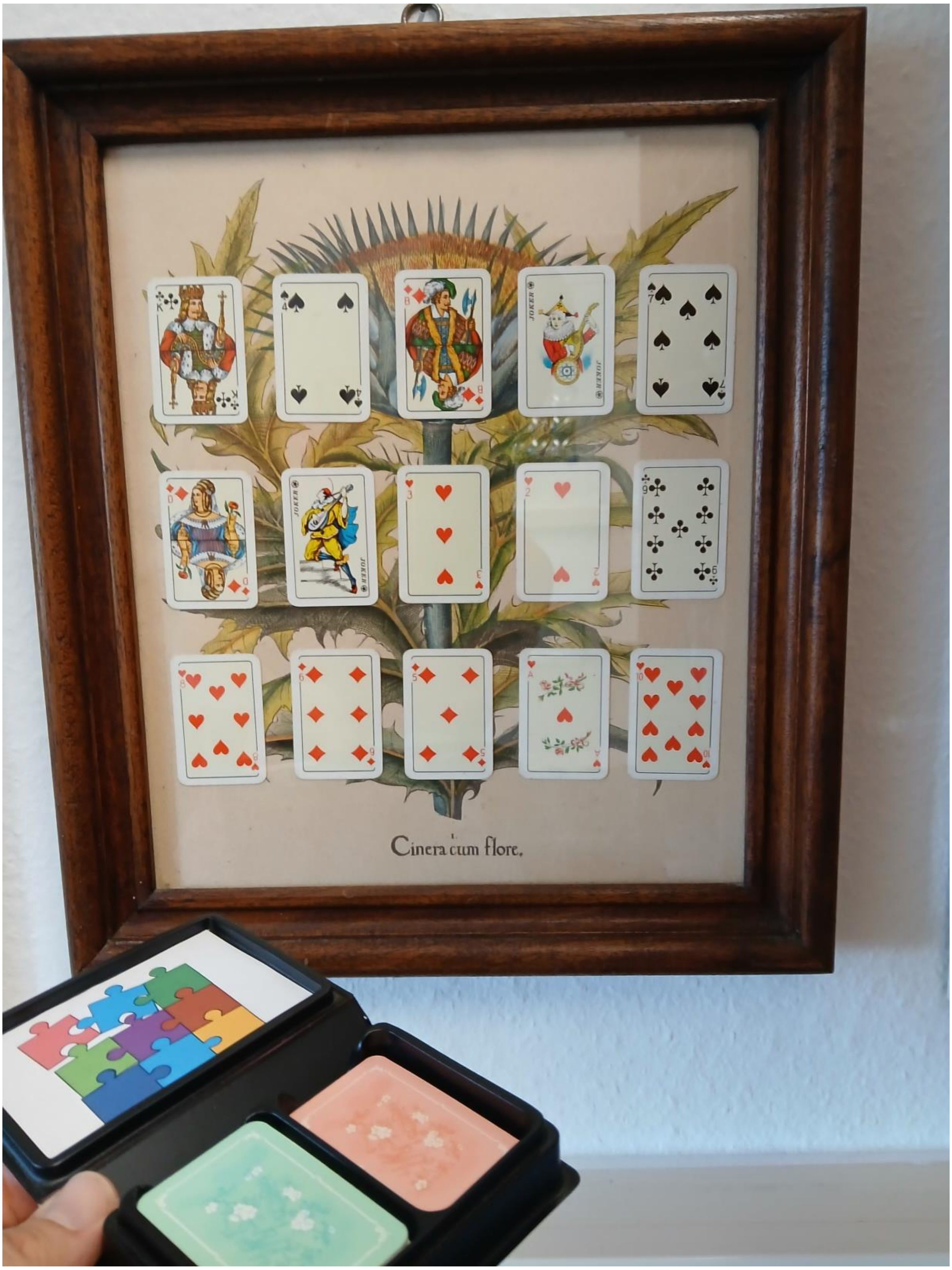

10 Knots

The players are allowed to tie 10 knots. Various ropes and wooden poles are available for this, see below. The instructions for the knots can be found on special cards. We used 50 knot cards from the publisher *Moses* 'Knotenbox' and selected 10. The instructions are supplemented by general terms (not shown due to copyright). Each knot is simple, whereby this puzzle corresponds well with the sewing school for veterinarians (surgery).

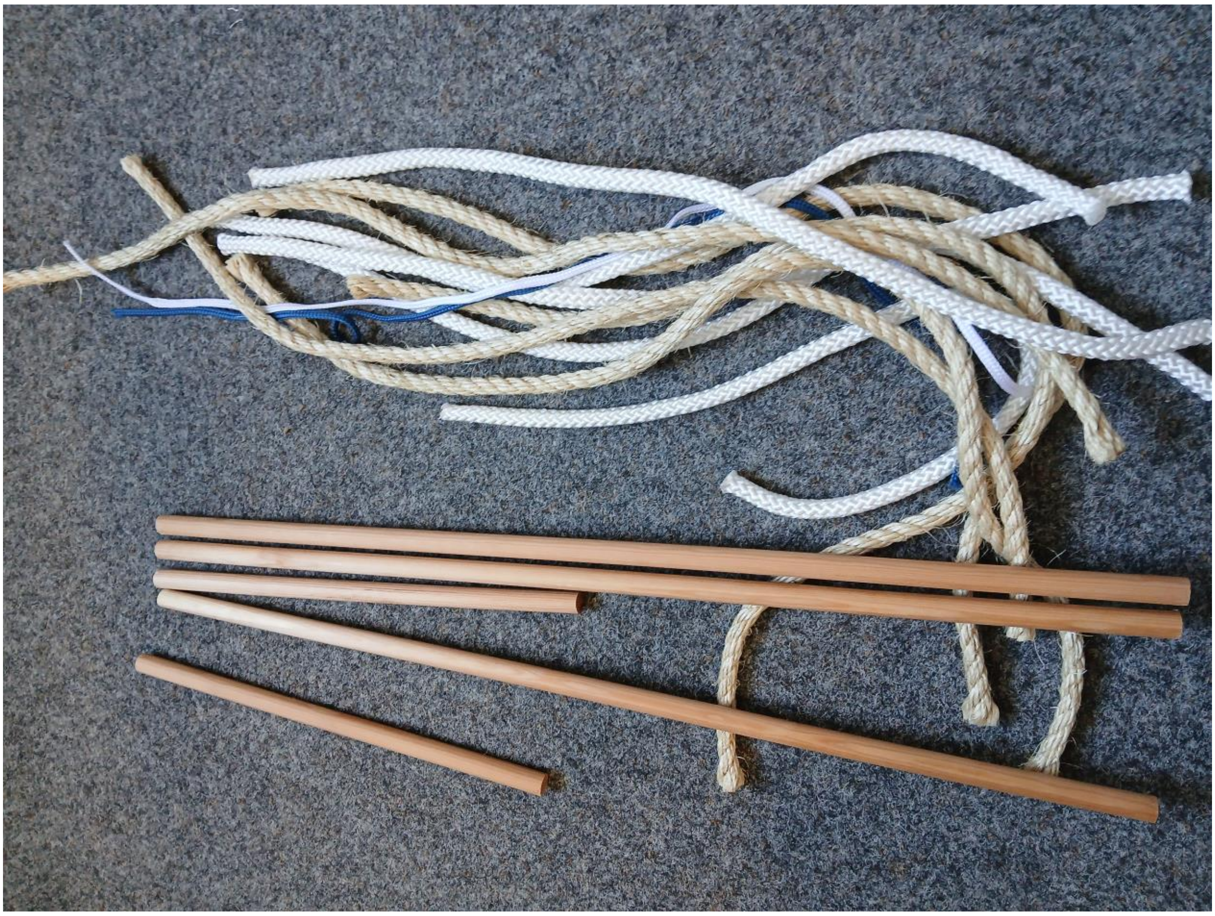

Magnet Blocks

The players have to assemble a snake analogue to the dog as physics puzzle. The snake is a little easier than the dog; for details, see main supplementary material of physics puzzle. Only the location of the purple stone is given and the outline of the snake (instructions not shown due to copyright).

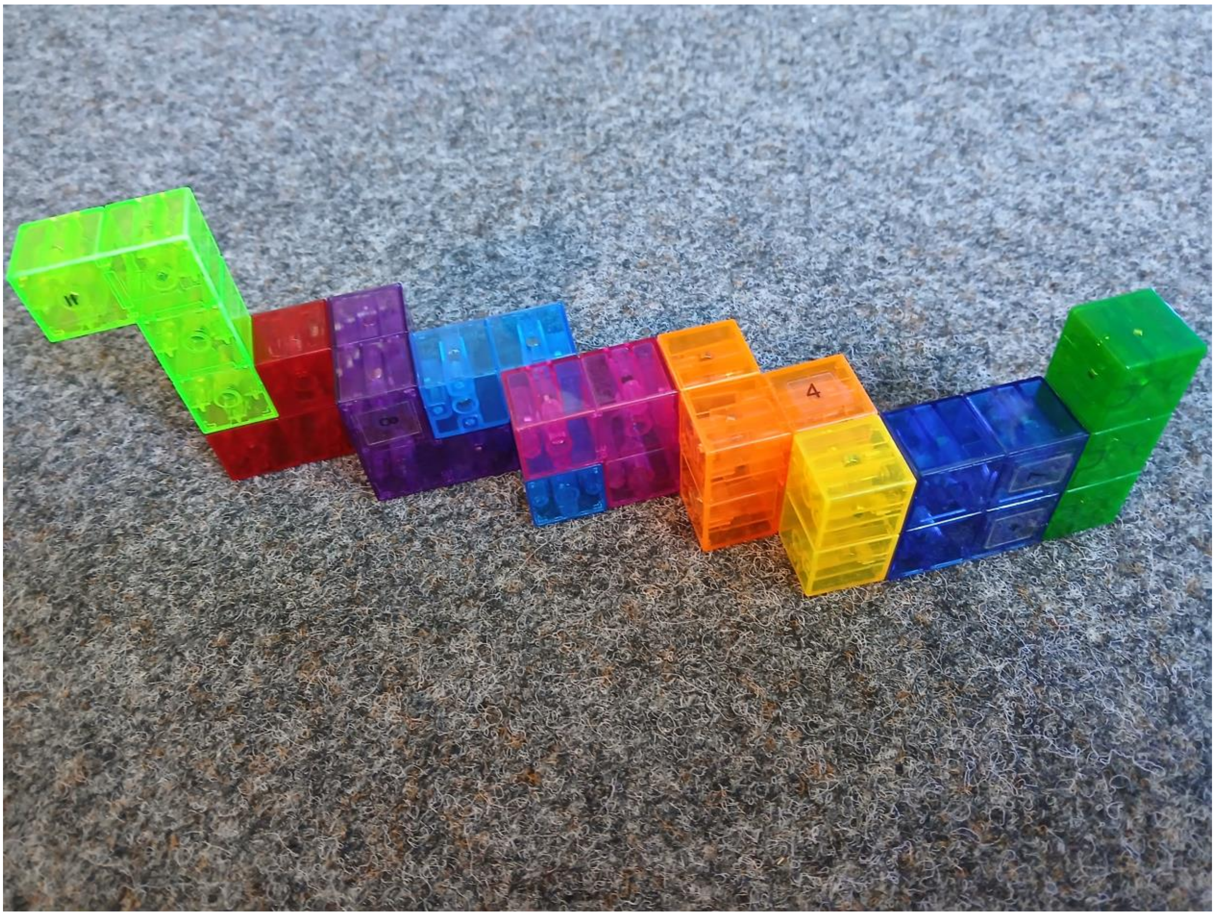

Periodic Table

The name of a physicist is derived directly from the name of the elements (first name and surname). The puzzle is moderately difficult, especially as there is a periodic table nearby.

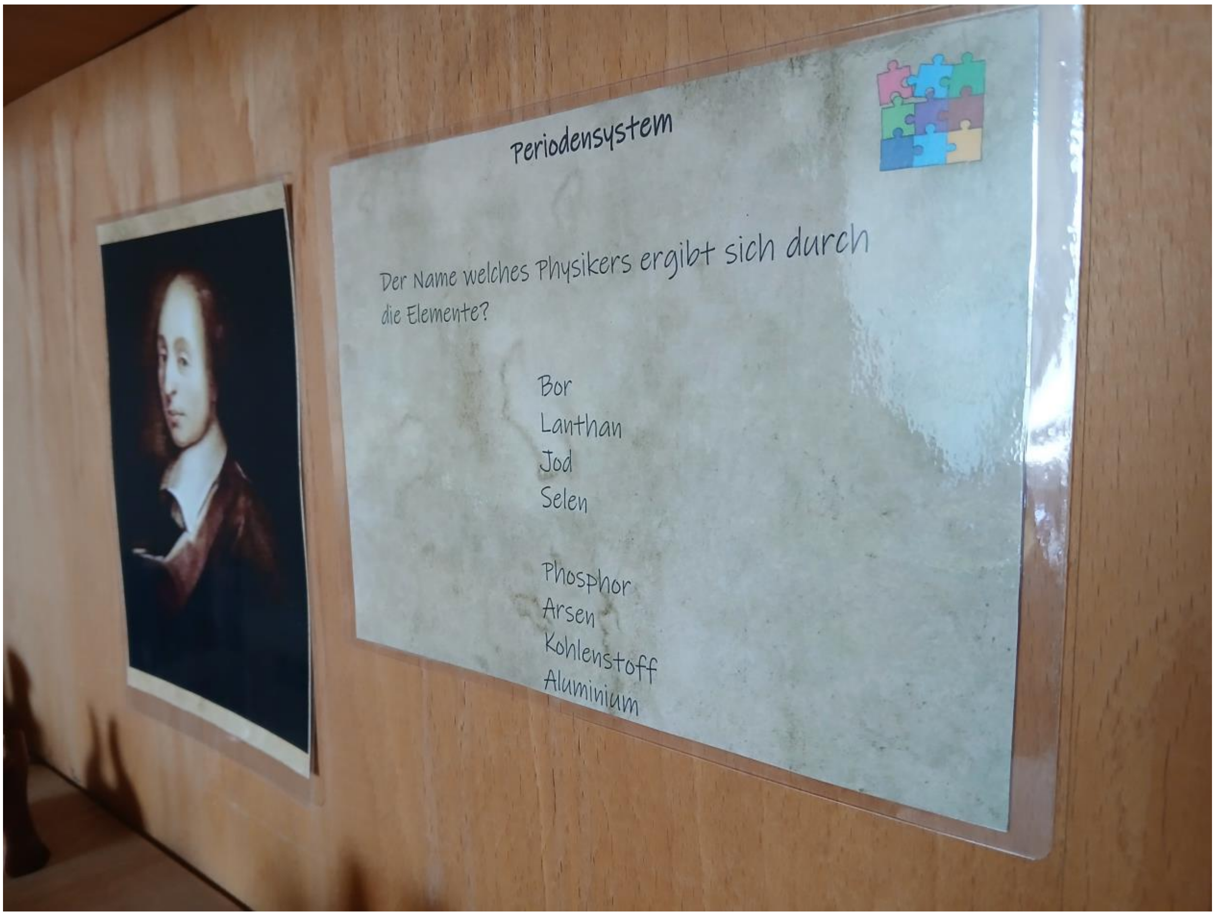

Wood bottle

You have to assemble a bottle from 3D wooden parts. The bottle was purchased from the company *Namesakes*. A small difficulty is that some parts look the same, but differ slightly from each other, making the assembly a small challenge.

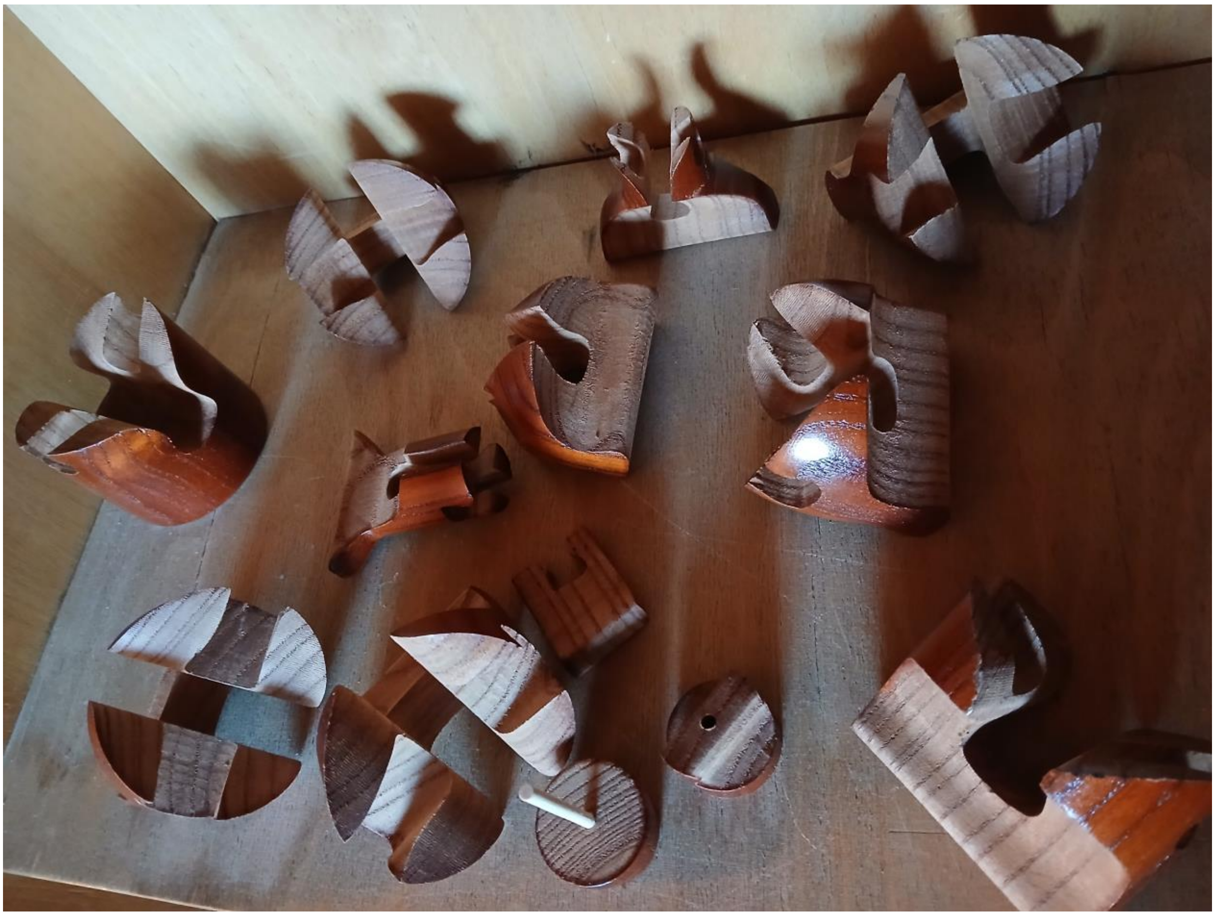

Chess

You can see a chess set with the initial moves of the Scandinavian defense. If you find the best moves alternately for black and white, the printed letters will give you the name of a physicist. The puzzle is self-designed and the level of difficulty is high.

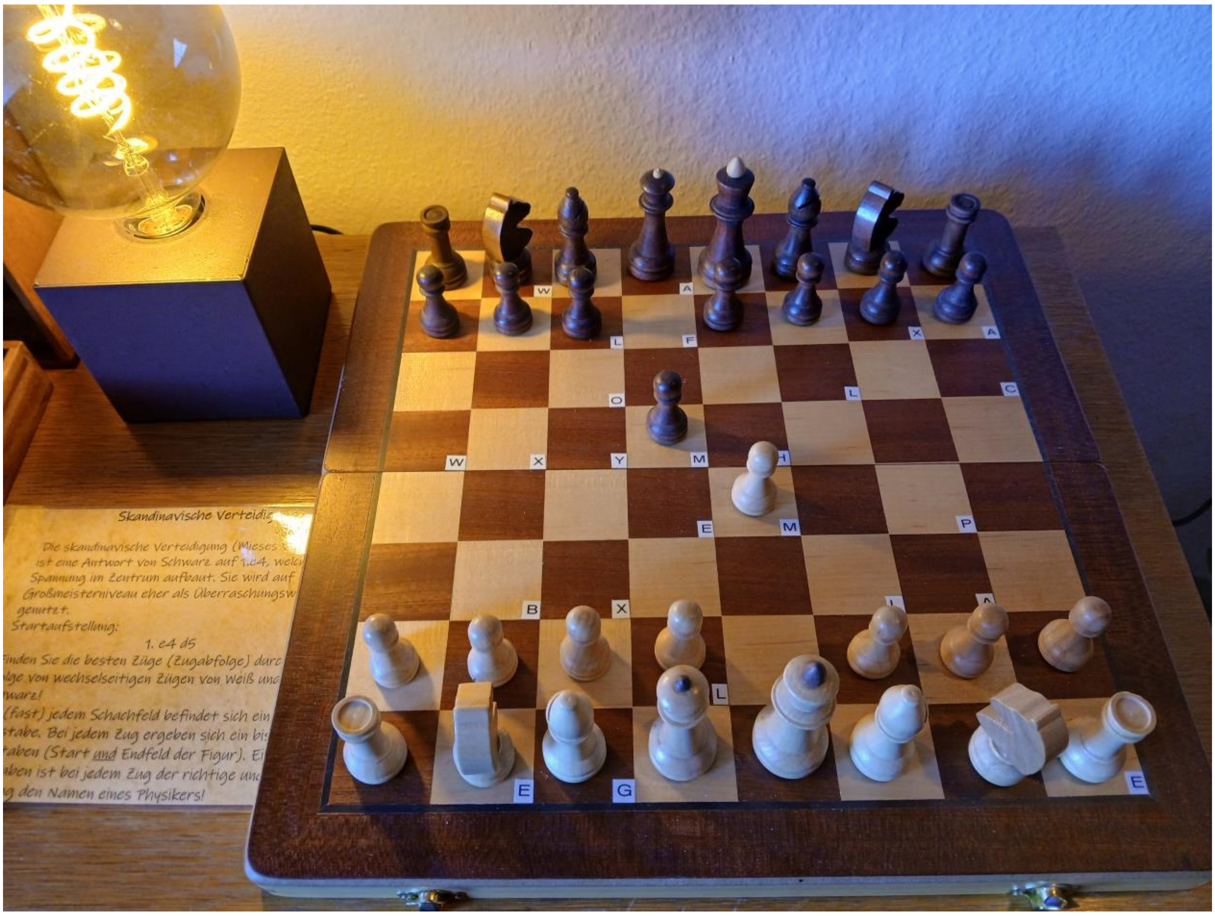

Quadratic Puzzle

The players have to assemble the puzzle from 9 pieces into a large 3x3 square, whereby the pictures of the physicists have to fit together. The difficulty is high; it trains frustration tolerance and is self-designed. It is homemade.

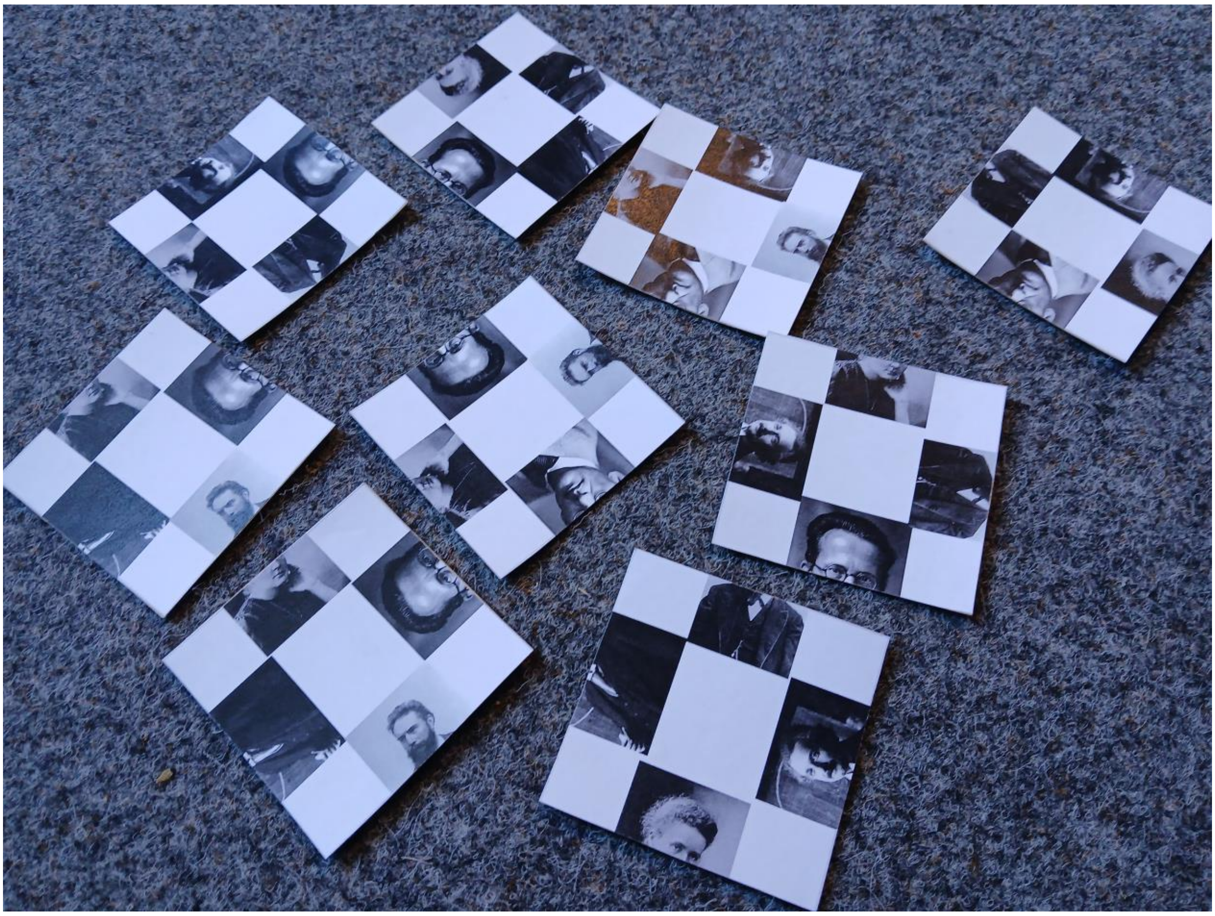

Text challenges: There are 5 text puzzles distributed in different boxes (together with the equipment of the physics puzzles). Example:

„Erwin Schrödingers Frühstück

Prof. Erwin Schrödinger ist ein schwieriger Mensch. Seine Frühstückseier müssen genau fünfzehn Minuten gekocht werden und keinen Augenblick kürzer. Heute hatte er für das Frühstück daheim keine Zeit. Er bittet Sie, ihm das Frühstück zu bereiten, doch die einzigen Uhren im Haus sind zwei Sanduhren.

Bei der größeren dauert es elf Minuten, bis der Sand durchgelaufen ist, bei der kleineren sieben Minuten. Was tun Sie? (Der Professor wird schon ungeduldig!)

Tipp: Überlegen Sie sich den ersten Schritt genau!“

Translated:

Erwin Schrödinger's breakfast

Prof Erwin Schrödinger is a difficult man. His breakfast eggs have to be cooked for exactly fifteen minutes and not a moment less. Today he did not have time for breakfast at home. He asks you to prepare breakfast for him, but the only clocks in the house are two hourglasses.

The larger one takes eleven minutes to run through the sand, the smaller one seven minutes. What do you do? (The professor is already getting impatient!)

Tip: Think carefully about the first step!

Hidden card: Easter Egg

A labelled business card is hidden in a secret compartment in the drawers in the room. It is known that this puzzle exists due to the instructions of the escape room, in which all Eurogame puzzles are listed. This puzzle is quite simple.

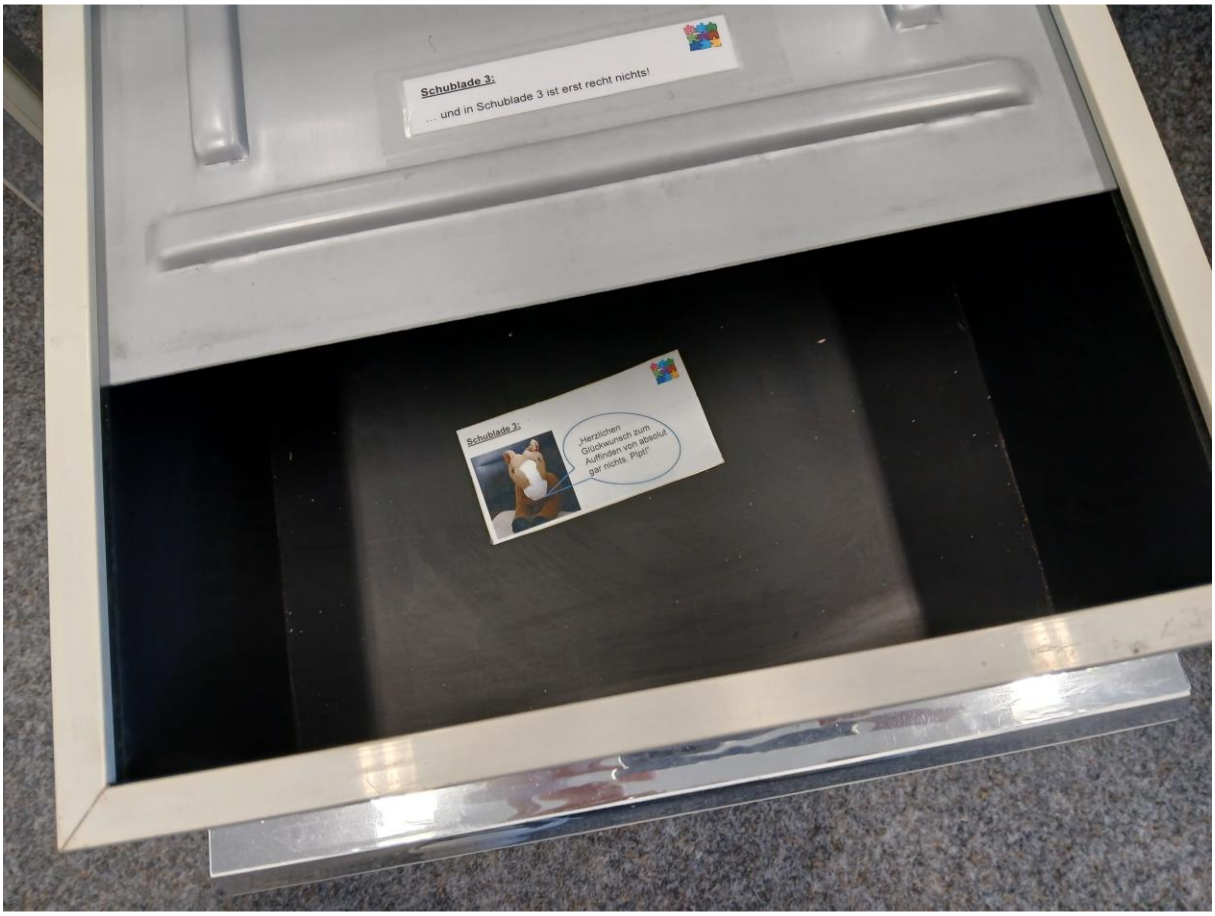

This is the original used questionnaire for the purpose to measure an increase in knowledge. Very specific questions were selected that could be answered with the help of the puzzles, the course of the game or the working materials. The corresponding discipline of physics is mentioned.

Escape-Room Evaluation/Wissenstransfer

Apple Puzzle: Forces

Ein Apfel wird dem Gravitationsfeld der Erde ausgesetzt.
Was gilt für den Apfel und die Erde nach dem 3. Newtonschen Gesetz?

- ☐ Nur die Erde zieht den Apfel an
- ☐ Apfel und Erde ziehen sich gegenseitig an
- ☐ Nur der Apfel zieht die Erde an
- ☐ Weiß ich nicht

Ferromagnetism

Wie heißt der Lichtrezeptor in den Augen, der für Vögel das Erdmagnetfeld sichtbar macht?

- ☐ Craptochrom
- ☐ Transchrom
- ☐ Cryptochrom
- ☐ Weiß ich nicht

Efficiency of Heat Engines

Was wissen Sie über den Wirkungsgrad η eines thermodynamischen Kreisprozesses?

- ☐ Es gilt $\eta = 1$.
- ☐ Es gilt immer $\eta < 1$.
- ☐ Es gilt immer $\eta > 1$
- ☐ Weiß ich nicht

X-Ray Absorption

So wie normales Licht wird auch Röntgenstrahlung durch Materie absorbiert.
Wie hängt die Absorption μ von Röntgenstrahlung zusammen mit der Ordnungszahl Z der Elemente?

- ☐ linear: $\mu \sim Z$
- ☐ quadratisch: $\mu \sim Z^2$
- ☐ kubisch: $\mu \sim Z^3$
- ☐ Weiß ich nicht

Electricity 50 Hz (ac)

Wie groß ist die Wechselstromfrequenz in den USA?

- ☐ 50 Hz
- ☐ 55 Hz
- ☐ 60 Hz
- ☐ Weiß ich nicht

Fluorescence

Wie bezeichnet man auch die Frequenz-Verschiebung bei Fluoreszenz?

- ☐ Planck-Shift
- ☐ Stokes-Shift
- ☐ Raman-Verschiebung
- ☐ Weiß ich nicht

Materials

Was wissen Sie über die elektrische Leitfähigkeit des Minerals Apatit (vereinfacht: $Ca_3(PO_4)_2$), das einen Hauptbestandteil von Knochen bildet?

- ☐ metallisch leitend
- ☐ Isolator
- ☐ Halbleiter
- ☐ Weiß ich nicht

Electric conductivity (dc)

Wie verhält sich der elektrische Widerstand R zur Querschnittsfläche A eines elektrisch leitenden Materials (z.B. Kupferkabel)?

- ☐ $R \sim A$
- ☐ $R \sim 1/A$
- ☐ $R \sim 1/A^2$
- ☐ Weiß ich nicht

Entropy

Welche Aussage zu den Eigenschaften der Entropie S ist richtig?

- ☐ S kann in einem Teilsystem zwar abnehmen, steigt dann aber in der Umgebung
- ☐ S kann im Gesamtsystem (= Summe aller Teilsysteme + Umgebung) sowohl zunehmen als auch abnehmen
- ☐ S kann in einem Teilsystem abnehmen, nimmt dann aber in der Umgebung auch ab
- ☐ Weiß ich nicht

Heat Engine

Was ist ein Stirlingmotor?

☐ Wärmeenergiemaschine ☐ Wärmekraftmaschine ☐ Kaltwärmemaschine ☐ Weiß ich nicht

Fridge: inverted Heat Engine

Welches dieser Geräte funktioniert nach dem (inversen) Prinzip des thermodynamischen Kreisprozesses?

☐ Heißluftfön ☐ Kühlschrank ☐ Inquisator ☐ Weiß ich nicht

Train Experiment: Kinematics

Was trifft auf Kreisbewegungen zu? Sie sind strenggenommen

☐ Bewegungen mit konstanter Geschwindigkeit ☐ beschleunigte Bewegungen ☐ Weiß ich nicht
